# The Discovery of Cyclic Lipopeptide Olenamidonins in a Deepsea-Derived *Streptomyces* Strain by Knocking Out a DtxR Family Regulator

**DOI:** 10.3390/md22060262

**Published:** 2024-06-06

**Authors:** Qiannan Sun, Dongqi Yu, Xueqing Zhang, Fei Xiao, Wenli Li

**Affiliations:** 1 Key Laboratory of Marine Drugs, Ministry of Education of China, School of Medicine and Pharmacy, Ocean University of China, Qingdao 266071, China; sunqiannan@stu.ouc.edu.cn (Q.S.); ydq1995ydq@163.com (D.Y.); zhangxq383@163.com (X.Z.); 2 Laboratory for Marine Drugs and Bioproducts, Qingdao National Laboratory for Marine Science and Technology, Qingdao 266000, China; 3 College of Chemistry & Pharmacy, Northwest A&F University, Yangling 712100, China

**Keywords:** deepsea-derived *Streptomyces*, silent gene cluster, DtxR family regulator, olenamidonins, gene disruption

## Abstract

Three new cyclic lipopeptides, olenamidonins A-C (**1**–**3**), in addition to two previously reported metabolites (**4** and **5**), were accumulated in the Δ*dtxR_so_* deletion mutant of deepsea-derived *Streptomyces olivaceus* SCSIO 1071. The structures of these cyclic lipopeptides were determined by a combination of spectroscopic methods and circular dichroism (CD) measurement. The antibacterial assay results showed that compounds **1–5** displayed different degrees of growth inhibition against multidrug-resistant (MDR) bacterial strains *Enterococcus faecalis* CCARM 5172 and *Enterococcus faecium* CCARM 5203 with minimum inhibitory concentrations (MICs) of 1.56−6.25 μg/mL.

## 1. Introduction

Microbial genome sequencing has unearthed uncountable cryptic secondary metabolite biosynthetic gene clusters (BGCs) in *Streptomyces*. However, most of them are silent under normal laboratory conditions [1]. Given that secondary metabolism is precisely controlled by a complex regulatory cascade network [2], the inactivation/overexpression of regulatory genes has been proven to be an effective way to unlock silent gene clusters [3].

DtxR (diphtheria toxin repressor) is a metal-dependent transcriptional regulator. DtxR family regulators, which were first identified in *Corynebacterium diphtheriae* [4], widely exist in high-GC content Gram-positive bacteria. They were found to be involved in the regulation of iron homeostasis, secondary metabolism, and morphological differentiation [5,6,7]. For example, the deletion of a DtxR family regulator gene *dmdR1* in *Streptomyces coelicolor* A3(2) led to a slow rate of spore formation and the loss of pigmented antibiotics undecylprodigiosin and actinorhodin production [8,9]. The DtxR family regulator IdeR from *Streptomyces avermitilis* could regulate the production of avermectin and oligomycin in a positive and negative way, respectively; additionally, its deletion resulted in a bald phenotype and delayed morphological differentiation in *S. avermitilis* [5].

**Figure 1:**
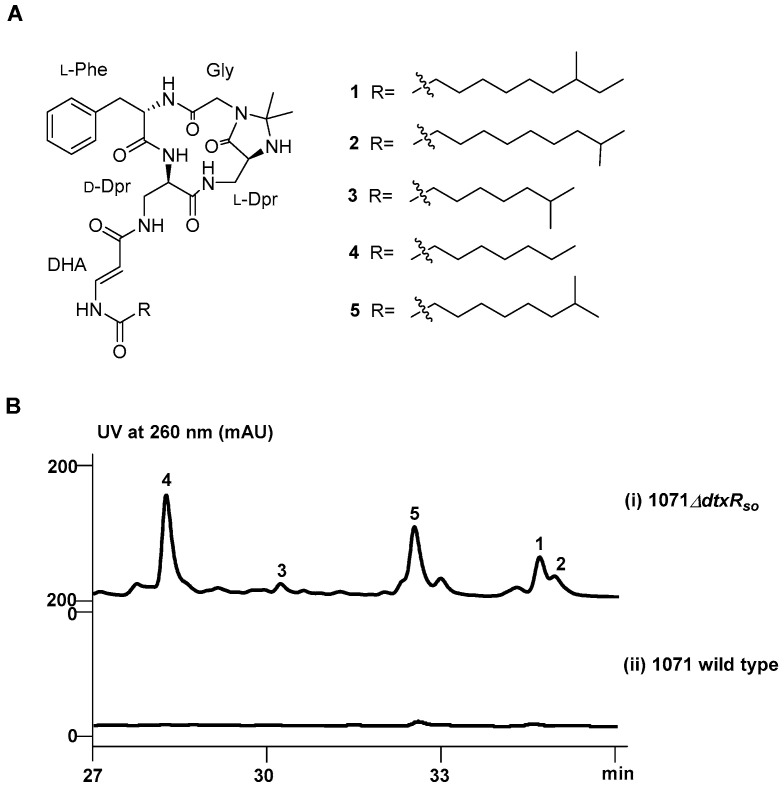
(**A**) Chemical structures of **1**–**5** and (**B**) HPLC analysis of Δ*dtxR_so_* (**i**) and wild-type (**ii**) strains.

In our efforts to discover novel natural products from deepsea-derived *Streptomyces* strains, pleiotropic regulators were set as target genes for genetic manipulation to activate silent BGCs. A DtxR family regulatory gene *dtxR_so_* was identified from the genome of deepsea-derived *Streptomyces olivaceus* SCSIO 1071. The inactivation of *dtxR_so_* led to the activation of cyclic lipopeptide compound olenamidonins, including three new (**1**–**3**) and two known (**4** and **5**) compounds (Figure 1A). The related structural analogs of olenamidonins, enamidonins, were reported to have a variety of bioactivities, including macrophage foam cell formation inhibition [10], acyl-CoA/cholesterol acyltransferase (ACAT) inhibition [11], and anti-Gram-positive bacteria activities [12]. Herein, we describe the isolation, structural identification, and antibacterial activity evaluation of these lipopeptides.

## 2. Results

The deepsea-derived *S. olivaceus* SCSIO 1071 was taxonomically classified through the phylogenetic analysis of its 16S rRNA gene sequence (Table S1, Figure S1) against the EzTaxon-e server Database. DtxR_so_ (Table S2) from *S. olivaceus* SCSIO 1071 showed 58.3% identity to DtxR (WP_010935052.1) from *C. diphtheriae* and is highly homologous to IdeR (BAC71567.1) from *S. avermitilis* (96.0% identity) and DmdR1 (CAC28070.1) from *S. coelicolor* (98.0% identity). While the deletion of *dtxR_so_* had no obvious impact on the growth and morphological differentiation, a series of new peaks were accumulated in the fermentation products of the Δ*dtxR_so_* mutant (Figure 1B, panel i) compared to those of the wild-type strain (Figure 1B, panel ii). 

**Figure 2:**
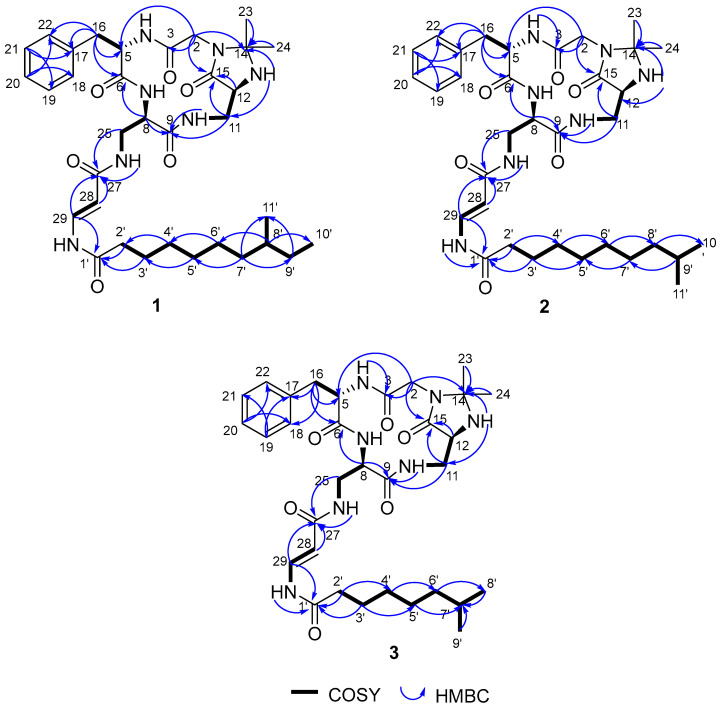
COSY and key HMBC correlations of **1**–**3**.

To elucidate the chemical structures of the activated compounds, a total volume of 35 L fermentation cultures of *S. olivaceus* SCSIO 1071Δ*dtxR_so_* was obtained, from which five compounds (**1**–**5**) were isolated. Their structures were identified via high-resolution electrospray ionization mass spectrometry (HRESIMS) and nuclear magnetic resonance (NMR) assignments. Compound **1** was isolated as a colorless oil. The molecular formula of **1** was determined to be C_34_H_51_N_7_O_6_ according to the HRESIMS data ([M + H]^+^ at *m*/*z* 654.3994, *calcd* 654.3979, Figure S6). The 1D and 2D NMR (HSQC, COSY, and HMBC) data showed the presence of amino acids phenylalanine (Phe), glycine (Gly), 2,3-diaminopropionic acid (Dpr), and a fatty acid moiety (Figure 2 and Figures S7–S12) [8]. HMBC correlations from H_2_-16 (Δ_H_ 3.07, 2.78) to C6 (Δ_C_ 171.1), C-17 (Δ_C_ 137.3), and C-18/22 (Δ_C_ 128.8), from H-20 (Δ_H_ 7.17) to C-18/22 (Δ_C_ 128.8), and from H-21 (Δ_H_ 7.24) to C-17 (Δ_C_ 137.3) and C-19 (Δ_C_ 128.3) confirmed the presence of a Phe residue. The HMBC correlations of the methylene signals H_2_-2 (Δ_H_ 4.17, 3.31) to C-3 (Δ_C_ 168.8) and C-5 (Δ_C_ 53.9) indicated the existence of the Gly residue linked at C-5 of Phe. Two Dpr units were established by COSY signals of H-10 (Δ_H_ 6.98)/H_2_-11 (Δ_H_ 3.50, 3.24)/H-12 (Δ_H_ 3.77)/H-13 (Δ_H_ 2.99) and H-7 (Δ_H_ 8.7)/H-8(Δ_H_ 4.36)/H_2_-25(Δ_H_ 3.31)/H-26 (Δ_H_ 7.77). The COSY correlations of H-28 (Δ_H_ 5.55)/H-29 (Δ_H_ 7.59) and the HMBC correlations from H-28 (Δ_H_ 5.55) and H-29 (Δ_H_ 7.59) to C-27 (Δ_C_ 166.6) confirmed the dehydro-β-alanine (DHA) unit. The ^1^H-^1^H coupling constant (^3^*J*_H-28, H-29_ =14.0 Hz) and the NOESY correlations for H-28 (Δ_H_ 5.55)/H-26 (Δ_H_ 7.77) defined the double-bond geometry as *28E*. The *N, N*-acetonide group was confirmed by the HMBC correlations from H_2_-2 (Δ_H_ 4.17, 3.31), H-13 (Δ_H_ 2.99), H_3_-23 (Δ_H_ 1.26), and H_3_-24 (Δ_H_ 1.18) to C-14 (Δ_C_ 76.3). Ten carbons of the C_11_ fatty acyl chain were identified by the COSY correlations, and the HMBC correlations from H_2_-7′ (Δ_H_ 1.27, 1.07) and H_2_-9′ (Δ_H_ 1.29, 1.08) to C-11′ (Δ_C_ 19.1) showed that the methyl group was attached to C-8′ (Δ_C_ 33.7). The advanced Marfey’s method [12] was used to determine the absolute configurations of the Phe and Dpr residues, and the result suggested the peptide backbone of compound **1** to be L-Phe-D-Dpr-L-Dpr-Gly (Figures S2 and S3). While the backbone was the same as those reported for autucedines and enamidonins [12,13], compound **1** contained a different 8′-methyldecanoyl side-chain. Therefore, compound **1** was identified as a new cyclic lipopeptide, named olenamidonin A. The ^1^H and ^13^C chemical shifts of **1** are listed in Table 1.

Compound **2** was isolated as a colorless oil. The molecular formula of **2** was determined to be C_34_H_51_N_7_O_6_ according to the HRESIMS data ([M + H]^+^ at *m*/*z* 654.3991, *calcd* 654.3979, Figure S13), which was the same as **1**. The NMR data showed that the backbone of **2** was the same as that of **1** (Figure 2 and Figures S14–S17). While **2** also contained a C_11_ fatty acyl chain, the COSY correlations of H_2_-8′ (Δ_H_ 1.49)/H-9′ (Δ_H_ 1.49)/H_3_-10′/11′ (Δ_H_ 0.84) combined with the HMBC correlations from H_2_-8′ (Δ_H_ 1.13) to C-10′/11′ (Δ_C_ 22.5) showed that the methyl group was attached to C-9′ (Δ_C_ 27.3) instead of C-8′ (Δ_C_ 33.7) compared with **1**. The CD spectra of **2** and **1** in MeOH display similar Cotton effects, supporting that **2** shares the same absolute configurations as **1** (Figure S4). Thus, compound **2** was identified as a new olenamidonin analog, named olenamidonin B. The ^1^H and ^13^C chemical shifts of **2** are listed in Table 1.

Compound **3** was isolated as a colorless oil. The molecular formula of **3** was determined to be C_32_H_47_N_7_O_6_ according to the HRESIMS data ([M + H]^+^ at *m*/*z* 626.3675, *calcd* 626.3666, Figure S18), which had two less methyl groups than **1**. The NMR data showed that the backbone of **3** was the same as **1** (Figure 2 and Figures S19–S23). Eight carbons of the C_9_ fatty acyl chain were identified by the COSY correlations, and two double-peak methyl signals with their HMBC correlations to C-7′ (Δ_C_ 27.3) confirmed that the methyl group was attached to C-7′ (Δ_C_ 27.3). The CD spectra of **3** and **1** in MeOH display similar Cotton effects, supporting that **3** shares the same absolute configurations as **1** (Figure S4). Compound **3** contained a different 7′-methyloctanoyl side-chain. Thus, compound **3** was identified as a new olenamidonin analog, named olenamidonin C. The ^1^H and ^13^C chemical shifts of **3** are listed in Table 1.

Compounds **4** and **5** were isolated as a colorless oil. The molecular formulas of **4** and **5** were determined to be C_31_H_45_N_7_O_6_ ([M + H]^+^ at *m*/*z* 612.3522, *calcd* 612.3510, Figure S24) and C_33_H_49_N_7_O_6_ ([M + H]^+^ at *m*/*z* 640.3840, *calcd* 640.3823, Figure S31), respectively. By comparing the NMR data (Figure 2 and Figures S25–S30 and S32–S37) with those reported in the literature, **4** and **5** were respectively confirmed to be known compounds autucedine A and autucedine D previously isolated from *Streptomyces olivaceus* SCSIO T05 [13], which is the same species as that of 1071 but is a different strain. The ^1^H and ^13^C NMR data for **4** and **5** are listed in Table S3. 

We further analyzed the olenamidonins’ BGC (Table S4) from 1071 and found that it is identical to the *aut* BGC from T05 (GenBank ID: WP_194276129.1-WP_194276149.1) [13] except a couple of nucleotides in between open reading frames. Based on the previously proposed biosynthetic pathway of autucedines [13], the 3-oxoacyl-ACP synthase OleH, the homolog of AutS, may display flexible substrate specificity, ligating different acyl-CoAs to DHA-ACP to finally generate lipopeptides with different lipid chains. While a molecular network analysis of autucedine analogs showed that AutS was able to use saturated/unsaturated lipochains with a length of C7 to C13, these compounds were not structurally characterized except autucedines A-E [13].

The antibacterial activities of compounds **1**–**5** were evaluated against three Gram-positive (*Micrococcus luteus* ML01, *Enterococcus faecalis* CCARM 5172, and *Enterococcus faecium* CCARM 5203) and two Gram-negative (*Escherichia coli* CCARM 1009 and *Pseudomonas aeruginosa* 15690) multidrug-resistant (MDR) bacterial strains. As shown in Table 2, while no antibacterial activities toward *M. luteus* ML01, *E. coli* CCARM 1009, or *P. aeruginosa* 15690 were detected for all the compounds, considerable growth inhibitions against *E. faecalis* CCARM 5172 and *E. faecium* CCARM 5203 were observed. Noticeably, compounds **1**–**2** and **5** exhibited stronger anti-*E. faecalis* CCARM 5172 activities (MICs = 1.56−3.12 μg/mL) than the positive control ciprofloxacin (MIC = 6.25 μg/mL), while **3**–**4** displayed comparable inhibition activities to that of ciprofloxacin. Compounds **1**–**2** and **5** also showed similar anti-*E. faecium* CCARM 5203 activities to that of the positive control tetracycline (MIC = 1.56 μg/mL), which were stronger than those of **3**–**4** (MIC = 3.12–6.25 μg/mL). The comparison of the structures and antibacterial activities of these compounds showed lipopeptides with C_11_- and C_10_-fatty acyl chains (**1**–**2**, **5**) have better inhibition potentials against *E. faecalis* CCARM 5172 and *E. faecium* CCARM 5203 than C_9_- and C_8_-fatty acyl chain analogs (**3**–**4**), suggesting that the length of the fatty acyl chain probably plays an important role in antibacterial activities.

**Figure 3:**
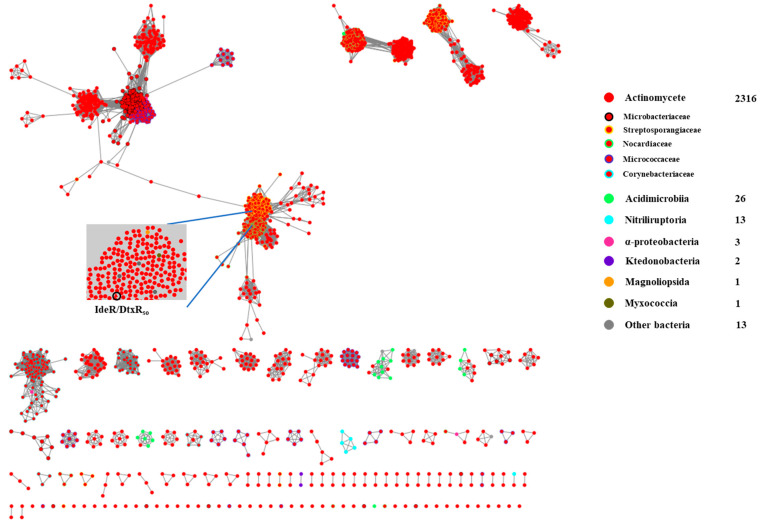
Distribution of DtxR_so_ homologs in bacteria. SSN analysis of DtxR_so_ homologs in diverse bacteria is based on cutoff E value of 10^−85^ and visualized by Cytoscape [14].

We further analyzed the distribution of the DtxR_so_ homologs across diverse bacteria. A sequence similarity network (SSN) analysis of 2375 DtxR_so_ homologs from the UniProt database was performed. As shown in Figure 3, these homologs mostly exist in *Actinomycetes* (in red), including families of *Microbacteriaceae*, *Streptosporangiaceae*, *Nocardiaceae*, *Micrococcaceae*, and *Corynebacteriaceae*. Noticeably, the *Acidimicrobiia* clade (in green) mainly has three paralogous groups, which are respectively associated with homolog(s) belonging to the *Actinomycetes* clade, suggesting their close relationship during evolution. Additionally, DtxR family regulators from *Magnoliopsida* (in orange) and *Myxococcia* (in dark green) are clustered with DtxR_so_/IdeR in the *Actinomycetes* clade, suggesting a possible horizontal transfer of the DtxR_so_ homologs across the bacterial world. Considering the massive cryptic secondary metabolite BGCs and the wide distribution of DtxR family regulators in *Actinomycetes*, the genetic manipulation of these regulators would be an alternative approach to unlock silent or cryptic gene clusters.

## 3. Conclusions

In this study, three new compounds, olenamidonins A-C (**1**–**3**), and two known compounds (**4** and **5**) were obtained from the Δ*dtxR_so_* mutant of deepsea-derived *S. olivaceus* SCSIO 1071. Compounds **1**–**5** showed different degrees of growth inhibitory activities against *E. faecalis* CCARM 5172 and *E. faecium* CCARM 5203. The comparison of their structures and antibacterial activities suggested that the length of the fatty acyl chain probably plays an important role in antibacterial activities. Feeding lipids with longer chains may contribute to the generation of olenamidonin derivatives with better activities. The activation of olenamidonin production by knocking out *dtxR_so_* is very intriguing, and the underlying regulatory mechanism is worthy of further investigation. Changing the fermentation conditions of Δ*dtxR_so_* could serve to investigate the impacts of the d*txR_so_* deletion on the productions of other metabolites. Given the wide distribution of DtxR_so_ homologs, they could serve as alternative targets for the activation of silent BGCs in actinomycetes strains.

## 4. Materials and Methods

### 4.1. General Materials and Methods

Bacterial strains and plasmids used in this study are listed in Table S1; primers are listed in Table S2. *Streptomyces olivaceus* SCSIO 1071 was isolated from a deepsea mud sample from the South China Sea. The NMR spectra were recorded on a Bruker Avance III 600. Chemical shifts were referenced to the residual DMSO-*d_6_* signal (Δ_H_ 2.50 and Δ_C_ 39.5 ppm for DMSO-*d_6_*). HRESIMS data were obtained on a Q-TOF Ultima Global GAA076 MS spectrometer. DNA isolation was carried out according to established protocols [15]. Plasmid was extracted using commercial kits (OMEGA).

### 4.2. Bioinformatic Analysis

The *ole* biosynthetic gene cluster was detected and analyzed using online antiSMASH 6.0.0 alpha software (http://antismash.secondarymetabolites.org/, 18 April 2024). Sequence comparisons and database searches were accomplished with BLAST programs (https://blast.ncbi.nlm.nih.gov/Blast.cgi, 18 April 2024).

The EFI-Enzyme Similarity Tool (EFI-EST) was used to generate sequence similarity networks (SSNs) [16]. For the distribution of DtxR_so_ homologs, the DtxR_so_ sequence was used as the query for searching a nonredundant protein sequence database using PSI-BLAST (position-specific iterated BLAST). A total of 2316 DtxR_so_ homologs were used to build the SSNs. The 10^−80^ SSN was generated by applying an E value cutoff of 10^−80^ to the full network. Each node in the network represents a single sequence, and each edge represents the pairwise connection between two sequences for which the BLASTP E value was lower than the cutoff value. SSNs were visualized and colored by Cytoscape (v3.7.2) [14].

### 4.3. The Construction of the dtxR_so_ Mutant Strain

A PCR-targeting strategy was adopted to obtain the *dtxR_so_* mutant strain [17,18]. Briefly, the amplified *aac(3)IV-oriT* resistance gene from pIJ773 was transformed into *E. coli* BW25113/pIJ790/pWLI615 to replace the *dtxR_so_* gene, resulting in the mutant cosmid pWLI1002 (Δ*dtxR_so_*). The mutant cosmid was passed through *E. coli* ET12567/pUZ8002 and then introduced into *S. olivaceus* SCSIO 1071 via conjugation [19]. The *dtxR_so_* mutant strain was selected through apramycin-resistant and neomycin-sensitive phenotype screening, followed by PCR confirmation.

### 4.4. Production and Purification of Olenamidonins

The fermentation of *S. olivaceus* SCSIO 1071/pWLI1002 in a total volume of 35 L was performed by growing cultures in 250 mL baffled Erlenmeyer flasks each containing 50 mL of AF/MS medium (glucose 20 g, yeast extract 2 g, soya flour 8 g, CaCO_3_ 4 g, NaCl 1 g, distilled H_2_O 1000 mL, pH = 7.3), incubated at 30 °C on a rotary shaker at 220 rpm for 7 days. The cells were extracted with acetone by sonication. The combined organic extracts were concentrated and then partitioned between 90% MeOH and *n*-hexane to yield two residues. The aqueous MeOH layer (1.05 g) was subjected to a reversed-phase (C18) open column chromatography with 20−100% MeOH to afford 6 fractions. Compounds **1**–**5** were obtained by a further purification of fraction 4 on reversed-phase HPLC (YMC-Pack ODS-A column 250 mm × 10 mm, i.d. 5 μm; wavelength at 260 nm) eluting with 49% CH_3_CN + 0.1% HCOOH (*v*/*v*) (1.5 mL/min).

Olenamidonin A (**1**): colorless oil; [α${]}_{D}^{25}$= +10.4 (c 0.05, MeOH); UV (MeOH) λ_max_ (log *ε*) 206 (3.03), 266 (3.04) nm; ^1^H and ^13^C NMR data, Table 1; HRESIMS *m/z* 654.3994 [M + H]^+^ (calcd for C_34_H_51_N_7_O_6_, 654.3979).

Olenamidonin B (**2**): colorless oil; [α${]}_{D}^{25}$= +34.6 (c 0.05, MeOH); UV (MeOH) λ_max_ (log *ε*) 206 (3.02), 266 (3.01) nm; ^1^H and ^13^C NMR data, Table 1; HRESIMS *m/z* 654.3991 [M + H]^+^ (calcd for C_34_H_51_N_7_O_6_, 654.3979).

Olenamidonin C (**3**): colorless oil; [α${]}_{D}^{25}$= +6.6 (c 0.05, MeOH); UV (MeOH) λ_max_ (log *ε*) 206 (3.42), 266 (3.40) nm; ^1^H and ^13^C NMR data, Table 1; HRESIMS *m/z* 626.3675 [M + H]^+^ (calcd for C_32_H_47_N_7_O_6_, 626.3666).

Autucedine A (**4**): colorless oil; [α${]}_{D}^{25}$= +22.4 (c 0.05, MeOH); UV (MeOH) λ_max_ (log *ε)* 208 (3.16), 266 (3.20) nm; ^1^H and ^13^C NMR data, Table S3; HRESIMS *m/z* 612.3522 [M + H]^+^ (calcd for C_31_H_45_N_7_O_6_, 612.3510).

Autucedine D (**5**): colorless oil; [α${]}_{D}^{25}$= +9 (c 0.05, MeOH); UV (MeOH) λ_max_ (log *ε*) 208 (3.22), 266 (3.27) nm; ^1^H and ^13^C NMR data, Table S3; HRESIMS *m/z* 640.3840 [M + H]^+^ (calcd for C_33_H_49_N_7_O_6_, 640.3823).

### 4.5. Antibacterial Assay

The MDR bacterial strains *M. luteus* ML01, *E. faecalis* CCARM 5172, *E. faecium* CCARM 5203, *E. coli CCARM* 1009, and *P. aeruginosa* 15690 were grown overnight at 37 °C in liquid LB medium, then diluted with the LB broth to 10^6^ CFU/mL. The sample solutions were diluted with MeOH to make a series of concentrations. After that, 20 μL of the sample solutions with different concentrations was dispensed into 180 μL of the bacterial suspension in 96-well plates and incubated at 37 °C for 18 h. The growth of MDR strains was measured on a microplate reader at a wavelength of 620 nm. Tetracycline (for *M. luteus* ML01, *E. faecium* CCARM 5203, *E. coli* CCARM 1009, and *P. aeruginosa* 15690) or ciprofloxacin (for *E. faecalis* CCARM 5172) was used as a positive control, methanol was used as a negative control, and LB broth was used as a blank.

## Figures and Tables

**Table 1:** ^1^H (600 MHz) and ^13^C (150 MHz) NMR data of **1–3** in DMSO-*d_6_*.

	**1**		**2**		**3**	
position	Δ_C_, type	Δ_H_ (*J* in Hz)	Δ_C_, type	Δ_H_ (*J* in Hz)	Δ_C_, type	Δ_H_ (*J* in Hz)
1-N						
2	44.6, CH_2_	4.17, d (14.3) 3.31, m	44.6, CH_2_	4.17, d (14.3) 3.31, m	44.6, CH_2_	4.17, d (14.4) 3.31, m
3	168.8, C		168.8, C		168.8, C	
4-NH		7.21, m		7.21, m		7.21, m
5	53.9, CH	4.49, td (8.8, 6.4)	53.9, CH	4.49, td (8.8, 6.4)	53.9, CH	4.49, td (8.8, 6.4)
6	171.1, C		171.1, C		171.1, C	
7-NH		8.70, d (7.7)		8.70, d (7.7)		8.70, d (7.7)
8	52.2, CH	4.36, q (7.3)	52.2, CH	4.36, q (7.3)	52.2, CH	4.36, q (7.3)
9	171.8, C		171.8, C		171.8, C	
10-NH		6.98, t (5.9)		6.98, t (5.9)		6.98, t (5.9)
11	38.9, CH_2_	3.50, m 3.24, m	38.9, CH_2_	3.50, m 3.24, m	38.9, CH_2_	3.50, m 3.24, m
12	58.9, CH	3.77, m	58.9, CH	3.77, d (8.4)	58.9, CH	3.77, d (8.2)
13-NH		2.99, d (9.4)		2.99, d (9.4)		2.99, d (9.4)
14	76.3, C		76.3, C		76.3, C	
15	172.8, C		172.8, C		172.8, C	
16	36.3, CH_2_	3.07, dd (13.9, 8.7) 2.78, dd (13.9, 6.2)	36.3, CH_2_	3.07, dd (13.9, 8.7) 2.78, dd (13.9, 6.2)	36.3, CH_2_	3.07, dd (13.9, 8.7) 2.78, dd (13.9, 6.2)
17	137.3, C		137.3, C		137.3, C	
18/22	128.8, CH	7.18, m	128.8, CH	7.18 m	128.8, CH	7.18, m
19/21	128.3, CH	7.24, m	128.3, CH	7.24 m	128.3, CH	7.24, m
20	126.3, CH	7.17, m	126.3, CH	7.17, m	126.3, CH	7.17, m
23	27.4, CH_3_	1.26, s	27.4, CH_3_	1.26, m	27.4, CH_3_	1.26, m
24	25.9, CH_3_	1.18, s	25.9, CH_3_	1.18, s	25.9, CH_3_	1.18, s
25	37.7, CH_2_	3.31, m	37.7, CH_2_	3.31, m	37.7, CH_2_	3.31, m
26-NH		7.77, t (5.7)		7.77, t (5.7)		7.77, t (5.7)
27	166.6, C		166.6, C		166.6, C	
28	103.5, CH	5.55, d (14.0)	103.5, CH	5.55, d (14.0)	103.5, CH	5.55, d (14.0)
29	133.8, CH	7.59, dd (13.9, 11.1)	133.8, CH	7.59, dd (13.9, 11.1)	133.8, CH	7.59, dd (13.9, 11.1)
30-NH		10.35, d (11.0)	NH	10.35, d (11.0)		10.35, d (11.0)
1′	171.5, C		171.5, C		171.5, C	
2′	35.4, CH_2_	2.24, t (7.3)	35.4, CH_2_	2.24, t (7.3)	35.3, CH_2_	2.24, t (7.3)
3′	24.7, CH_2_	1.54, m	24.7, CH_2_	1.54, m	24.8, CH_2_	1.54, m
4′	28.6, CH_2_	1.25, m	28.6, CH_2_	1.25, m	28.8, CH_2_	1.25, m
5′	29.1, CH_2_	1.26, m	28.8, CH_2_	1.24, m	26.4, CH_2_	1.26, m
6′	26.3, CH_2_	1.24, m	29.2, CH_2_	1.23, m	38.3, CH_2_	1.15, m
7′	35.9, CH_2_	1.25, m 1.07, m	26.8, CH_2_	1.24, m	27.3, CH	1.49, m
8′	33.7, CH	1.27, m	38.4, CH_2_	1.13, m	22.48, CH_3_	0.84, d (6.6)
9′	28.9, CH_2_	1.29, m 1.08, m	27.3, CH	1.49, m	22.48, CH_3_	0.84, d (6.6)
10′	11.2, CH_3_	0.83, m	22.5, CH_3_	0.84, d (6.6)		
11′	19.1, CH_3_	0.82, m	22.5, CH_3_	0.84, d (6.6)		

**Table 2:** Antibacterial activities of compounds **1**–**5** against MDR bacterial strains (MIC, μg/mL).

Strains	MIC (μg/mL)						
	1	2	3	4	5	Tetracycline	Ciprofloxacin
*M. luteus* ML01	>50	>50	>50	>50	>50	6.25	
*E. faecalis* CCARM 5172	3.12	1.56	6.25	6.25	3.12		6.25
*E. faecium* CCARM 5203	1.56	1.56	3.12	6.25	1.56	1.56	
*E. coli* CCARM 1009	>50	>50	>50	>50	>50	1.56	
*P. aeruginosa* 15690	>50	>50	>50	>50	>50	12.5	

## Data Availability

The data are included in the article and the Supplementary Materials.

## Supplementary Materials

The following supporting information can be downloaded at https://www.mdpi.com/article/10.3390/md22060262/s1, Figure S1: Neighbor-joining phylogenetic tree of *S. olivaceus* 1071 SCSIO; Figures S2 and S3: The HPLC chromatogram data of D-FDAA derivatives of the amino acid units in olenamidonin A (**1**); Figures S4–S37: HR-ESI-MS, ^1^H NMR, ^13^C NMR and, HSQC, HMBC, ^1^H-^1^H COSY, NOESY, ECD spectra of compounds **1**–**5**. Tables S1 and S2: 1490 nt 16S rRNA and *dtxRso* sequence of *S. olivaceus* SCSIO 1071; Tables S3 and S4: Bacteria, plasmids and primer pairs used in this study; Table S5: ^1^H (600 MHz) and ^13^C (150 MHz) NMR data of **4** and **5**; Table S6: Proposed functions of proteins encoded by the *ole* biosynthetic gene cluster in *S. olivaceus* SCSIO 1071. Reference [20] are cited in the Supplementary Materials.
