# Supplementary material for: The Discovery of Cyclic Lipopeptide Olenamidonins in a Deepsea-Derived Streptomyces Strain by Knocking Out a DtxR Family Regulator
Source: Mar Drugs. 2024 Jun 6;22(6):262. doi: 10.3390/md22060262 (PMC11204786; doi:10.3390/md22060262)
Supplement: Supplementary file 1 [file marinedrugs-22-00262-s001.zip › marinedrugs-2997071-supplementary.pdf]

Supporting Information

The Discovery of Cyclic Lipopeptide Olenamidonins in a Deepsea-Derived *Streptomyces* Strain by Knocking out a DtxR Family Regulator

Qiannan Sun[1], Dongqi Yu[1], Xueqing Zhang[1], Fei Xiao[1]*, Wenli Li[1,2,3]*

* Author to whom correspondence should be addressed

Fei Xiao Email: xiaofei3450@ouc.edu.cn

Wenli Li Email: liwenli@ouc.edu.cn

[1]Key Laboratory of Marine Drugs, Ministry of Education of China, School of Medicine and Pharmacy, Ocean University of China, Qingdao, China

[2]Laboratory for Marine Drugs and Bioproducts, Qingdao National Laboratory for Marine Science and Technology, Qingdao, China

[3]College of Chemistry & Pharmacy, Northwest A&F University, Yangling, China

Table of contents

Table S1. A 1490 nt 16S rRNA sequence of *S. olivaceus* SCSIO 1071. 4

Table S2. *dtxR*$_{so}$ sequence of *S. olivaceus* SCSIO 1071. 5

Table S3. Bacteria and plasmids used in this study. 5

Table S4. The primer pairs used in this study[a]. 6

Table S5. ^{1}H (600 MHz) and ^{13}C (150 MHz) NMR data of **4** and **5** in DMSO-*d6*. 7

Table S6. The proposed functions of proteins encoded by the *ole* biosynthetic gene cluster in *S. olivaceus* SCSIO 1071. 8

Figure S1. A neighbor-joining phylogenetic tree of the *S. olivaceus* 1071 SCSIO based on 16S rDNA sequences. 9

Figure S2. HPLC chromatogram data of D-FDAA derivatives of amino acid units in olenamidonin A (**1**). 9

Figure S3. An advanced Marfey's analysis of the D-FDLA-derivatized Phe-Dpr dipeptide. 10

Figure S4. ECD spectra of **1**-**5** in MeOH. 11

Figure S5. Inactivation of *dtxR*$_{so}$. 12

Figure S6. HR-ESIMS spectrum of **1**. 12

Figure S7. ^{1}H NMR spectrum of **1** in DMSO-d_6. 13

Figure S8. ^{13}C NMR spectrum of **1** in DMSO-d_6. 13

Figure S9. HSQC spectrum of **1** in DMSO-d_6. 14

Figure S10. ^{1}H-^{1}H COSY spectrum of **1** in DMSO-d_6. 14

Figure S11. HMBC spectrum of **1** in DMSO-d_6. 15

Figure S12. NOESY spectrum of **1** in DMSO-d_6. 15

Figure S13. HR-ESIMS spectrum of **2**. 16

Figure S14. ^{1}H NMR spectrum of **2** in DMSO-d_6. 16

Figure S15. HSQC spectrum of **2** in DMSO-d_6. 17

Figure S16. ^{1}H-^{1}H COSY spectrum of **2** in DMSO-d_6. 17

Figure S17. HMBC spectrum of **2** in DMSO-d_6. 18

Figure S18. HR-ESIMS spectrum of **3**. 18

Figure S19. ^{1}H NMR spectrum of **3** in DMSO-d_6. 19

Figure S20. HSQC spectrum of **3** in DMSO-d_6. 19

Figure S21. ^{1}H-^{1}H COSY spectrum of **3** in DMSO-d_6. 20

Figure S22. HMBC spectrum of **3** in DMSO-d_6. 20

Figure S23. NOESY spectrum of **3** in DMSO-d_6. 21

Figure S24. HR-ESIMS spectrum of **4**. 21

Figure S25. ^{1}H NMR spectrum of **4** in DMSO-d_6. 22

Figure S26. ^{13}C NMR spectrum of **4** in DMSO-d_6. 22

Figure S27. HSQC spectrum of **4** in DMSO-d_6. 23
Figure S28. ^{1}H-^{1}H COSY spectrum of **4** in DMSO-d_6. 23
Figure S29. HMBC spectrum of **4** in DMSO-d_6. 24
Figure S30. NOESY spectrum of **4** in DMSO-d_6. 24
Figure S31. HR-ESIMS spectrum of **5**. 25
Figure S32. ^{1}H NMR spectrum of **5** in DMSO-d_6. 25
Figure S33. ^{13}C NMR spectrum of **5** in DMSO-d_6. 26
Figure S34. HSQC spectrum of **5** in DMSO-d_6. 26
Figure S35. ^{1}H-^{1}H COSY spectrum of **5** in DMSO-d_6. 27
Figure S36. HMBC spectrum of **5** in DMSO-d_6. 27
Figure S37. NOESY spectrum of **5** in DMSO-d_6. 28

Table S1. A 1490 nt 16S rRNA sequence of *S. olivaceus* SCSIO 1071.

***S. olivaceus* SCSIO 1071**	**Sequence**
16S rRNA	AGAGTTTGATCCTGGCTCAGGACGAACGCTGGCGGCGTG<br>CTTAACACATGCAAGTCGAACGATGAACCACTTCGGTGGG<br>GATTAGTGGCGAACGGGTGAGTAACACGTGGGCAATCTG<br>CCCTGCACTCTGGGACAAGCCCTGGAAACGGGGTCTAAT<br>ACCGGATATTGATCTTCACGGGCATCTGTGAGGTTCGAAA<br>GCTCCGGCGGTGCAGGATGAGCCCGCGGCCTATCAGCTT<br>GTTGGTGAGGTAATGGCTCACCAAGGCGACGACGGGTAG<br>CCGGCCTGAGAGGGCGACCGGCCACACTGGGACTGAGA<br>CACGGCCCAGACTCCTACGGGAGGCAGCAGTGGGGAAT<br>ATTGCACAATGGGCGAAAGCCTGATGCAGCGACGCCGCG<br>TGAGGGATGACGGCCTTCGGGTTGTAAACCTCTTTCAGC<br>AGGGAAGAAGCGAAAGTGACGGTACCTGCAGAAGAAGC<br>GCCGGCTAACTACGTGCCAGCAGCCGCGGTAATACGTAG<br>GGCGCAAGCGTTGTCCGGAATTATTGGGCGTAAAGAGCT<br>CGTAGGCGGCTTGTCACGTCGGTTGTGAAAGCCCGGGG<br>CTTAACCCCGGGTCTGCAGTCGATACGGGCAGGCTAGAG<br>TTCGGTAGGGGAGATCGGAATTCCTGGTGTAGCGGTGAA<br>ATGCGCAGATATCAGGAGGAACACCGGTGGCGAAGGCG<br>GATCTCTGGGCCGATACTGACGCTGAGGAGCGAAAGCGT<br>GGGGAGCGAACAGGATTAGATACCCTGGTAGTCCACGCC<br>GTAAACGGTGGGCACTAGGTGTGGGCAACATTCCACGTT<br>GTCCGTGCCGCAGCTAACGCATTAAGTGCCCCGCCTGGG<br>GAGTACGGCCGCAAGGCTAAAACTCAAAGGAATTGACGG<br>GGGCCCGCACAAGCGGCGGAGCATGTGGCTTAATTCGAC<br>GCAACGCGAAGAACCTTACCAAGGCTTGACATACACCGG<br>AAACGGCCAGAGATGGTCGCCCCCTTGTGGTCGGTGTAC<br>AGGTGGTGCATGGCTGTCGTCAGCTCGTGTCGTGAGATG<br>TTGGGTTAAGTCCCGCAACGAGCGCAACCCTTGTCCCGT<br>GTTGCCAGCAAGCCCCTTCGGGGGTGTTGGGGACTCAC<br>GGGAGACCGCCGGGGTCAACTCGGAGGAAGGTGGGGA<br>CGACGTCAAGTCATCATGCCCCTTATGTCTTGGGCTGCAC<br>ACGTGCTACAATGGCCGGTACAATGAGCTGCGATACCGCA<br>AGGTGGAGCGAATCTCAAAAAGCCGGTCTCAGTTCGGAT<br>TGGGGTCTGCAACTCGACCCCATGAAGTCGGAGTCGCTA<br>GTAATCGCAGATCAGCATTGCTGCGGTGAATACGTTCCCG<br>GGCCTTGTACACACCGCCCGTCACGTCACGAAAGTCGGT<br>AACACCCGAAGCCGGTGGCCCAACCCCTTGTGGGAGGG<br>AGCTGTCGAAGGTGGGACTGGCGATTGGGACGAAGTCG<br>TAACAAGGTAGCC

Table S2. *dtxR*$_{so}$ sequence of *S. olivaceus* SCSIO 1071.

***S. olivaceus* SCSIO 1071**	**Sequence**
dtxR$_{so}$	ATGTCCGGACTGATCGACACCACGGAGATGTACCTCCGC<br>ACCATCCTGGAGCTGGAGGAGGAAGGTGTGGTCCCGATG<br>CGCGCCCGGATCGCCGAGCGGCTCGACCAGAGCGGCCC<br>GACGGTCAGTCAGACGGTGGCGCGCATGGAGCGCGACG<br>GTCTGGTGTCCGTGGCGGCCGACCGGCACCTGGAGCTG<br>ACCGAGGAGGGCAGGCGGCTGGCGACGCGCGTGATGC<br>GCAAGCACCGGCTCGCGGAGTGCCTGCTCGTCGACGTG<br>ATCGGCCTGGAGTGGGAGCAGGTGCACGCCGAGGCGTG<br>CCGCTGGGAGCACGTGATGAGCGAGGCCGTCGAGCGCC<br>GCGTGCTCGAGCTGCTGCGCCACCCGACCGAGTCGCCG<br>TACGGGAACCCGATTCCGGGCCTGGAGGAGCTGGGGGA<br>GACGGACATCGCCGACCCGTTCCTGGACGAGGGCATGG<br>TCTCGCTCGCCGACCTCGACCCGGGCATGGAGGGCAAG<br>ACGGTCGTCGTGCGGCGGATCGGCGAGCCGATCCAGAC<br>GGACGCGCAGCTGATGTACACGCTGCGCCGGGCGGGTG<br>TGCAGCCCGGTTCGGTGGTGAGCGTGACCGAGTCGGCG<br>GGCGGCGTGCTGGTGGGCAGTGGCGGCGAGGCGGCCG<br>AGCTGGAGTCGGACACCGCCTCCCACGTGTTCGTGGCC<br>AAGCGCTGA

Table S3. Bacteria and plasmids used in this study.

**Strains or plasmids**	**Description**	**Reference or source**
Strains		
E. coli DH5α	Host strain for general cloning	Stratagene
E. coli ET12567/pUZ8002	Host strain for conjugation	[1]
E. coli BW25113/pIJ790	Host strain for PCR-targeting	[2]
Streptomyces olivaceus SCSIO 1071	Wild-type strain	This study
Plasmids		
pIJ773	Apr^R, source of *acc(3)IV-oriT* cassette	[1]
pIJ790	Cm^R, λ RED recombination plasmid	[1]
pWLI1001	Genomic library cosmid harboring the *dtxR*$_{so}$ gene	This study
pWLI1002	pWLI1001 derivative where *dtxR*$_{so}$ was replaced with *acc(3)IV-oriT* cassette	This study

Table S4. The primer pairs used in this study[a].

Primers	Sequence (5'→3')
Knockout	
ΔdtxR$_{so}$-MF	ATGTCCGGACTGATCGACACCACGGAGATGTACCTCCGCATTCCGGGGATCCGTCGACC
ΔdtxR$_{so}$-MR	TCAGCGCTTGGCCACGAACACGTGGGAGGCGGTGTCCGATGTAGGCTGGAGCTGCTTC
Confirmation of the mutants	
ΔdtxR$_{so}$-CF	GGGCACGGTTGTCGGTGGTA
ΔdtxR$_{so}$-CR	GCCAGGAGCCCCTTCGTCAT

[a]Underlined letters represent nucleotides homologous to the DNA regions internal to target genes.

Table S5. ^{1}H (600 MHz) and ^{13}C (150 MHz) NMR data of **4** and **5** in DMSO-*d6*.

	**4**		**5**	
Position	δ_C, type	δ_H (J in Hz)	δ_C, type	δ_H (J in Hz)
1-N				
2	44.6, CH_2	4.17, d (14.3)<br>3.31, m	44.6, CH_2	4.17, d (14.3)<br>3.31, m
3	168.8, C		168.8, C	
4-NH		7.21, m		7.21, m
5	53.9, CH	4.49, td (8.8, 6.4)	53.9, CH	4.49, td (8.8, 6.4)
6	171.1, C		171.1, C	
7-NH		8.71, d (7.7)		8.71, d (7.7)
8	52.2, CH	4.36, q (7.3)	52.2, CH	4.36, q (7.3)
9	171.8, C		171.8, C	
10-NH		6.98, t (5.9)		6.98, t (5.9)
11	38.9, CH_2	3.50, dd (13.7, 6.2)<br>3.24, dd (10.4, 5.0)	38.9, CH_2	3.50, dd (13.7, 6.2)<br>3.24, dd (10.4, 5.0)
12	58.9, CH	3.77, d (8.4)	58.9, CH	3.77, d (8.4)
13-NH		2.99, d (9.4)		2.99, d (9.4)
14	76.3, C		76.3, C	
15	172.8, C		172.8, C	
16	36.3, CH_2	3.07, dd (13.9, 8.7)<br>2.78, dd (13.9, 6.2)	36.3, CH_2	3.07, dd (13.9, 8.7)<br>2.78, dd (13.9, 6.2)
17	137.3, C		137.3, C	
18/22	128.8, CH	7.18, m	128.8, CH	7.18, m
19/21	128.3, CH	7.24, m	128.3, CH	7.24, m
20	126.3, CH	7.17, m	126.3, CH	7.17, m
23	27.4, CH_3	1.26, m	27.4, CH_3	1.26, m
24	25.9, CH_3	1.18, s	25.9, CH_3	1.18, s
25	37.7, CH_2	3.31, m	37.7, CH_2	3.31, m
26-NH		7.77, t (5.7)		7.77, t (5.7)
27	166.6, C		166.6, C	
28	103.5, CH	5.55, d (14.0)	103.5, CH	5.55, d (14.0)
29	133.8, CH	7.59, dd (13.9, 11.1)	133.8, CH	7.59, dd (13.9, 11.1)
30-NH		10.35, d (11.0)		10.35, d (11.0)
1′	171.5, C		171.5, C	
2′	35.4, CH_2	2.24, t (7.3)	35.3, CH_2	2.24, t (7.3)
3′	24.7, CH_2	1.54, m	24.7, CH_2	1.54, m
4′	28.4, CH_2	1.25, m	28.6, CH_2	1.25, m
5′	28.5, CH_2	1.24, m	29.0, CH_2	1.24, m
6′	31.1, CH_2	1.24, m	26.7, CH_2	1.26, m
7′	22.0, CH_2	1.26,	38.4, CH_2	1.13, m
8′	13.9, CH_3	0.89, t (7.0)	27.4, CH	1.49, m
9′			22.5, CH_3	0.84, d
10′			22.5, CH_3	0.84, d

[a]Signals were overlapped with other signals.

Table S6. The proposed functions of proteins encoded by the *ole* biosynthetic gene cluster in *S. olivaceus* SCSIO 1071.

Protein	Size (aa)	Proposed function	Homolog	Identity/ Similarity (%)
-1	518	Carboxylesterase	Carboxylesterase (MDA4890073.1), *Streptomyces* sp. MS2A	84/89
OleA	438	Histidine kinase	SceX (ANH11419.1), *Streptomyces* sp. SD85	45/58
OleB	727	Transporter	Transporter (WP_103817670.1), *Streptomyces* sp. Ru62	50/68
OleC	68	MbtH family protein	Tsk13 (UMM61374.1), *Streptomyces tasikensis* P46	59/74
OleD	317	ABC transporter	Atr29 (QBG38790.1), *Streptomyces atratus* SCSIO ZH16	58/73
OleE	245	4'-phosphopantetheinyl transferase	AsuC1(AIL50185.1), *Streptomyces aureus*	36/48
OleF	275	SARP family transcriptional regulator	SARP family transcriptional regulator (BAX64243.1), *Streptomyces* sp. MSC090213JE08	45/60
OleG	344	ornithine cyclodeaminase	ornithine cyclodeaminase (WP_130881053.1), *Streptomyces syringium*	58/73
OleH	309	3-oxoacyl-ACP synthase	3-oxoacyl-ACP synthase (KUN30549.1), *Streptomyces corchorusii*	53/70
OleI	480	ATP-grasp protein	ATP-grasp protein (QCW80778.1), *Streptomyces* sp. S6	61/70
OleJ	427	Decarboxylase	EtmH (QSI97707.1), *Streptomyces achromogenes* subsp. *streptozoticus*	57/69
OleK	92	Acyl carrier protein	EtmI (QSI97708.1), *Streptomyces achromogenes* subsp. *streptozoticus*	43/67
OleL	296	Hypothetical protein	Hypothetical protein (OEV19204.1), *Streptomyces nanshensis*	36/46
OleM	234	Acyl-CoA dehydrogenase	EtmK (QSI97710.1), *Streptomyces achromogenes* subsp. *streptozoticus*	34/46
OleN	387	Acyl-CoA dehydrogenase	acyl-CoA dehydrogenase (WP_311120300.1), *Streptomyces durocortorensis*	57/71
OleO	214	Transcriptional regulator	Transcriptional regulator (WP_150259904.1), *Streptomyces venezuelae*	75/87
OleP	278	2,3-diaminopropionate biosynthesis protein	2,3-diaminopropionate biosynthesis protein (WP_274911273.1), *Streptomyces* sp. WZ-12	68/77
OleQ	2183	Non-ribosomal peptide synthetase	Atr21 (QBG38782.1), *Streptomyces atratus*	38/50
OleR	2416	Non-ribosomal peptide synthetase	Atr22 (QBG38783.1), *Streptomyces atratus*	48/59
OleS	273	ABC transporter	Atr30 (QBG38791.1), *Streptomyces atratus*	42/61
OleT	187	Hypothetical protein	Hypothetical protein (GGW99313.1), *Streptomyces malachitofuscus*	83/87
OleU	195	TetR family transcriptional regulator	TetR family transcriptional regulator (NEC45849.1), *Streptomyces* sp. SID8016	82/88
OleV	168	DinB family protein	DinB family protein (WP_186776788.1), *Streptomyces salinarius*	80/89
OleW	208	SAM-dependent methyltransferase	SAM-dependent methyltransferase (MDG9694720.1), *Streptomyces* sp. DH17	87/93
OleX	194	XRE family transcriptional regulator	XRE family transcriptional regulator (AUO16412.1), *Streptomyces* sp. IMB7-145	62/72
OleY	418	DUF4190 domain-containing protein	DUF4190 domain-containing protein (MZE69516.1), *Streptomyces* sp. SID5789	80/87
OleZ	250	GntR family transcriptional regulator	GntR family transcriptional regulator (MCF2125887.1), *Streptomyces* sp. STD 3.1	96/98
+1	61	SPOR domain-containing protein	SPOR domain-containing protein (WP_055418037.1), *Streptomyces pactum*	88/91
+2	144	Hydrolase	Hydrolase (ADM46379.1), *Streptomyces* sp. CS	66/73

Note: The *ole* BGC is almost identical to the *aut* BGC (GenBank ID: WP_194276129.1-WP_194276149).

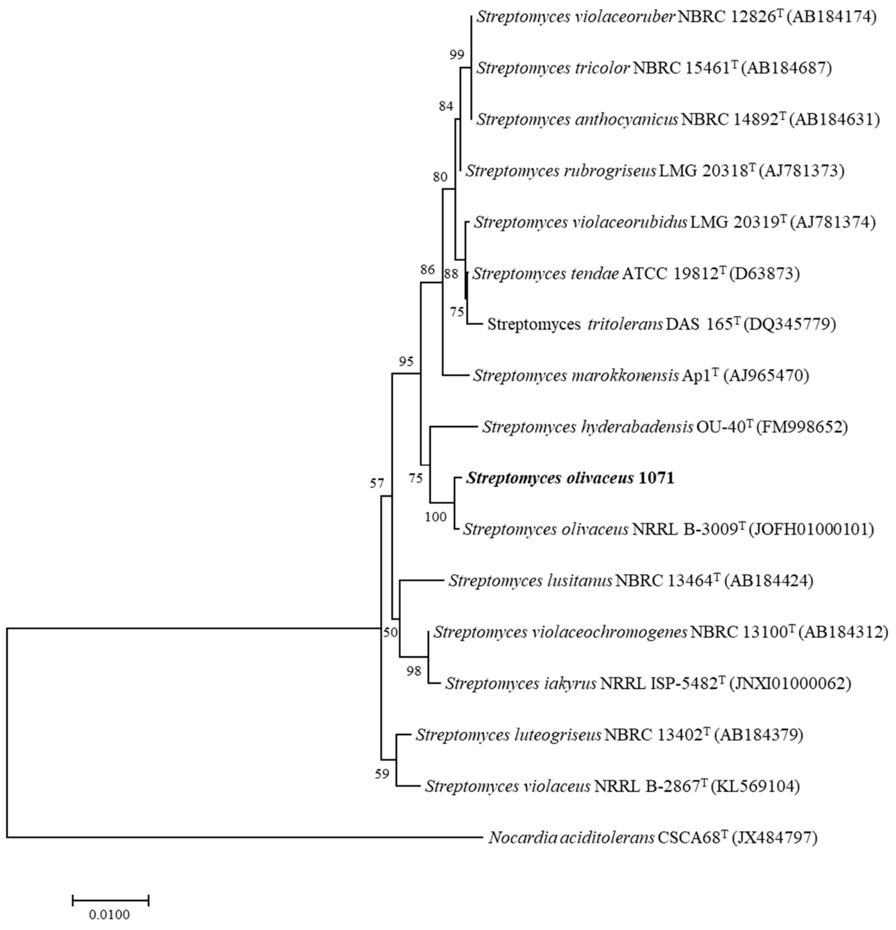

Figure S1. A neighbor-joining phylogenetic tree of the *S. olivaceus* SCSIO 1071 based on 16S rDNA sequences.

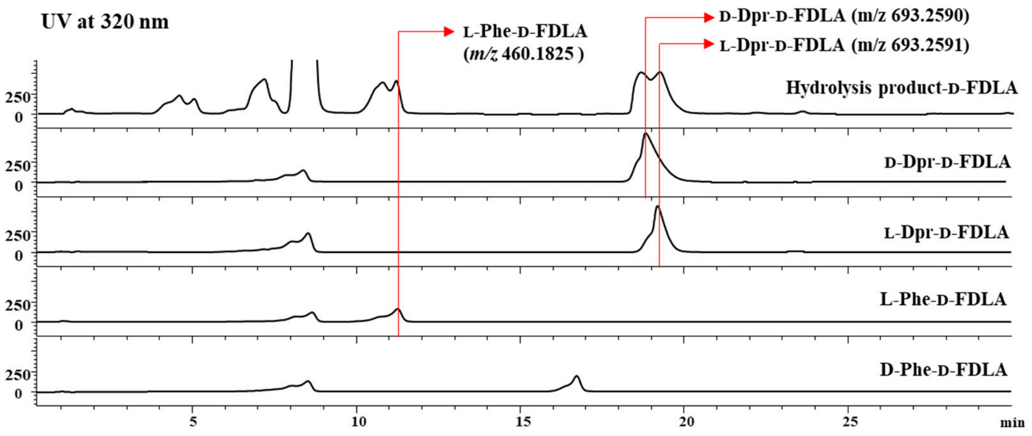

Figure S2. HPLC chromatogram data of D-FDAA derivatives of amino acid units in olenamidonin A (**1**).

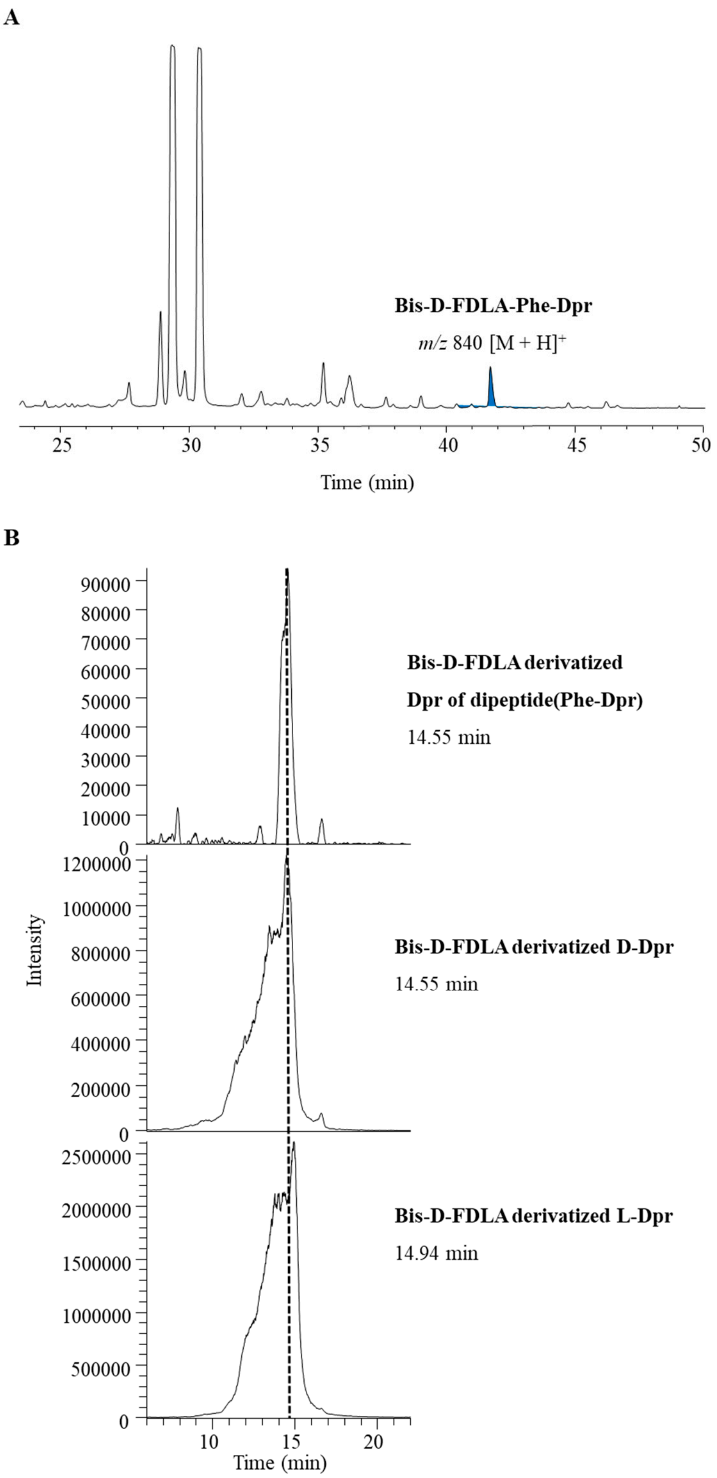

Figure S3. An advanced Marfey's analysis of the D-FDLA-derivatized Phe-Dpr dipeptide. (A) An HPLC chromatogram of the partial hydrolysate of **1** derivatized with D-FDLA. The peak for bis-D-FDLA-Phe-Dpr is colored in blue. (B) Extracted ion chromatograms at *m/z* 693 $[M+H]^+$ of the D-FDLA-derivatized Dpr of the dipeptide and D-FDLA-derivatized authentic D- and L-Dpr.

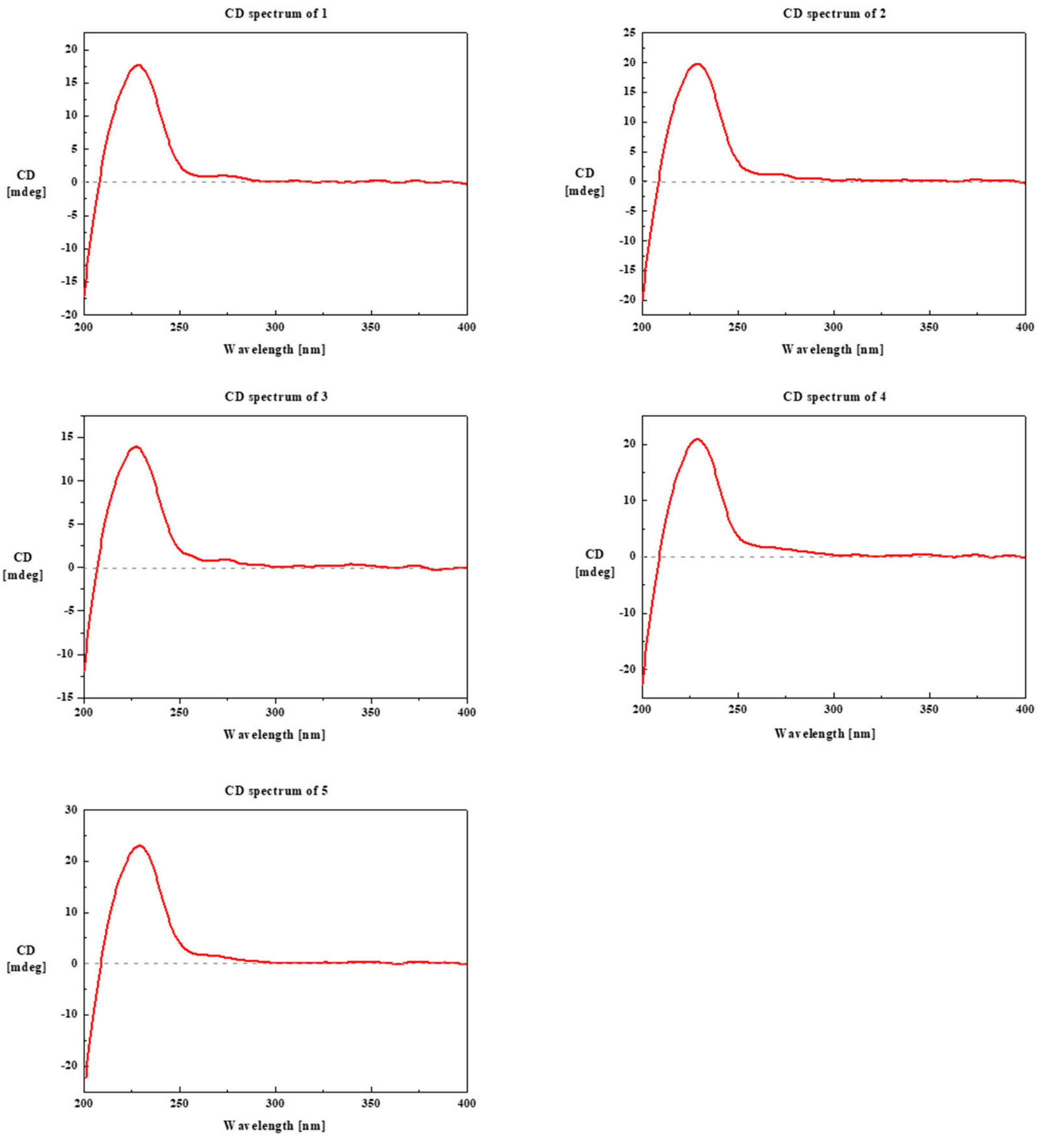

Figure S4. ECD spectra of **1**-**5** in MeOH**.**

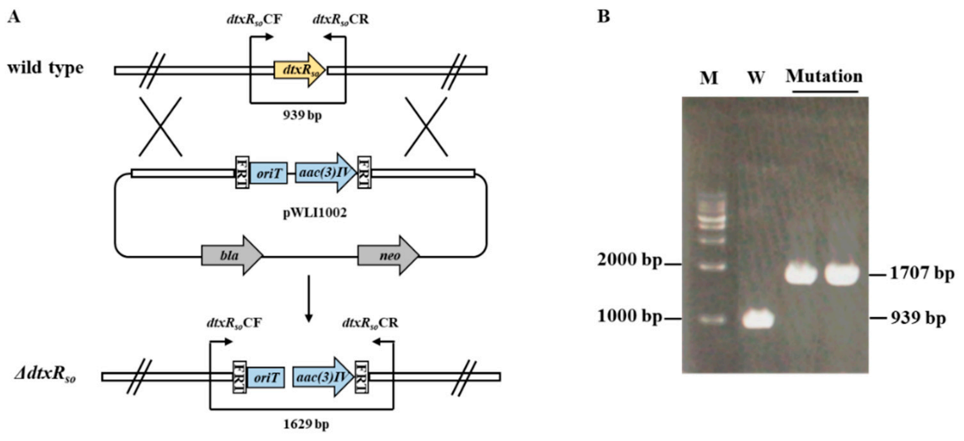

Figure S5. Inactivation of *dtxR*$_{so}$. (A) Construction of *dtxR*$_{so}$ inactivation mutant. (B) PCR

confirmation of double-crossover mutant. M: 1 kb DNA marker; W: *S. olivaceus* SCSIO 1071 wild-type strain; M: *$dtxR_{so}$* gene inactivation mutant.

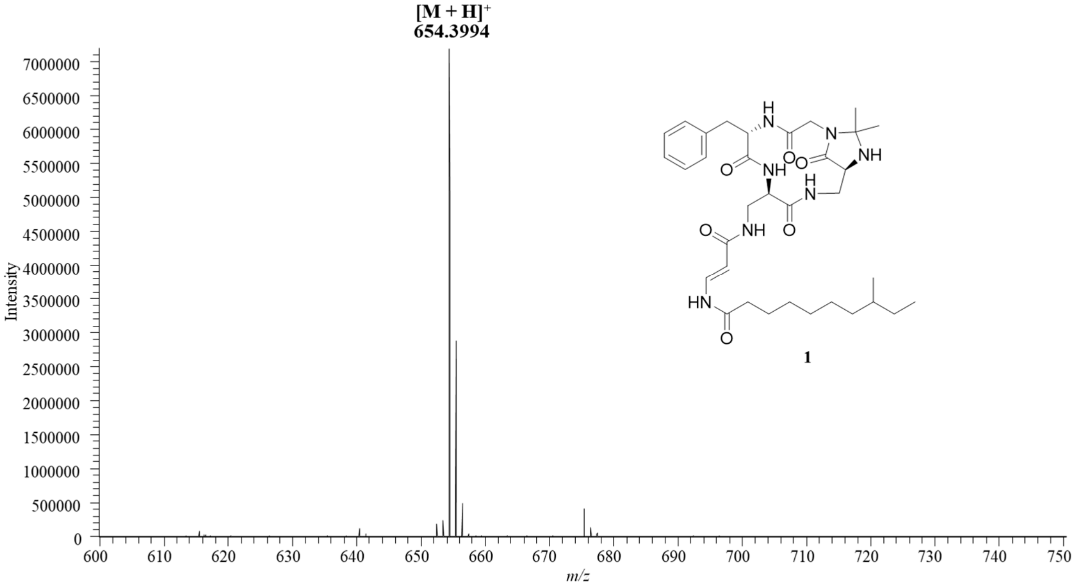

Figure S6. HRESIMS spectrum of **1**.

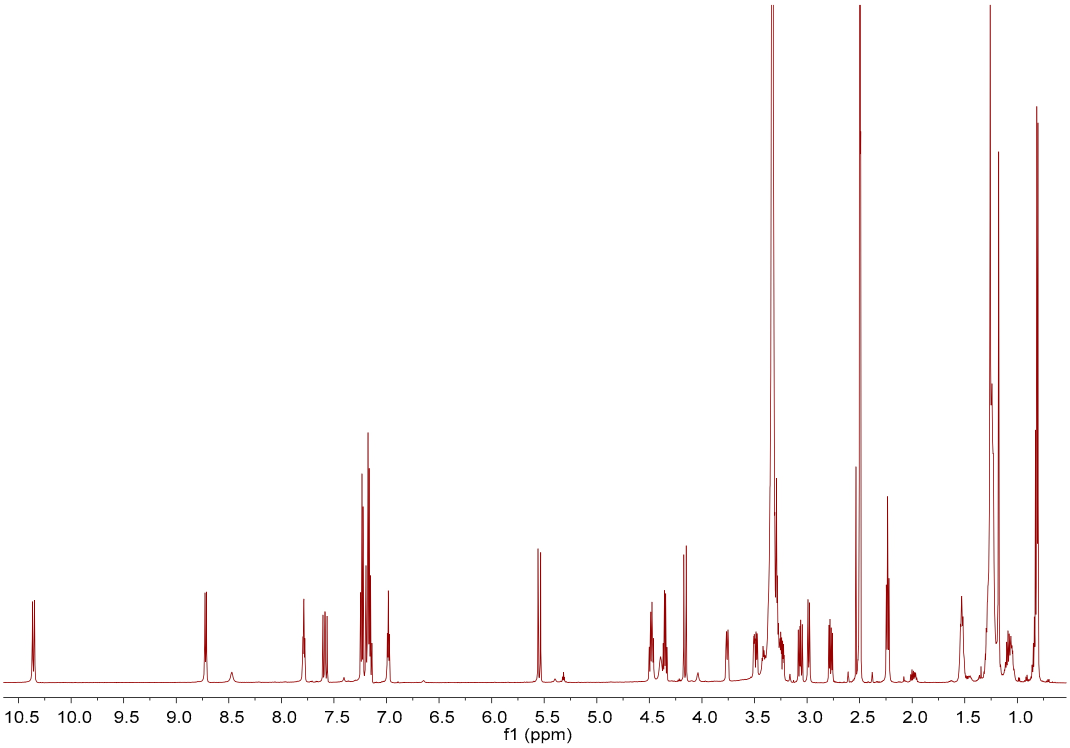

Figure S7. ^{1}H NMR spectrum of **1** in DMSO-d_6.

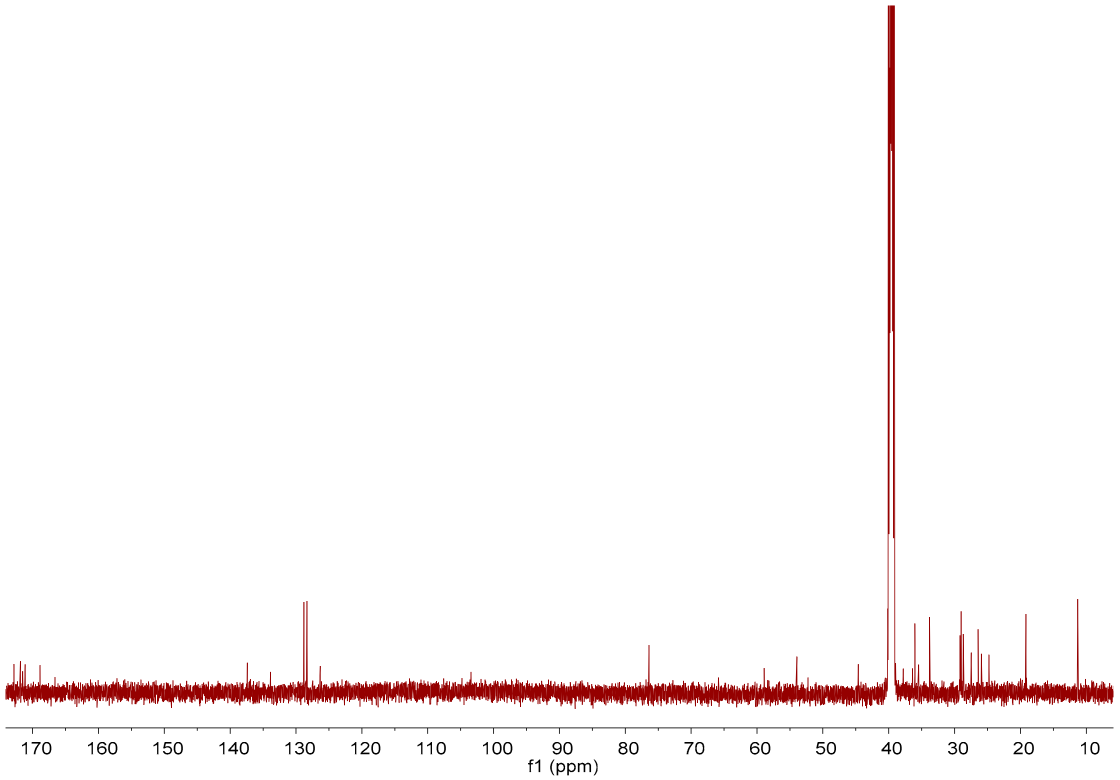

Figure S8. ^{13}C NMR spectrum of **1** in DMSO-d_6.

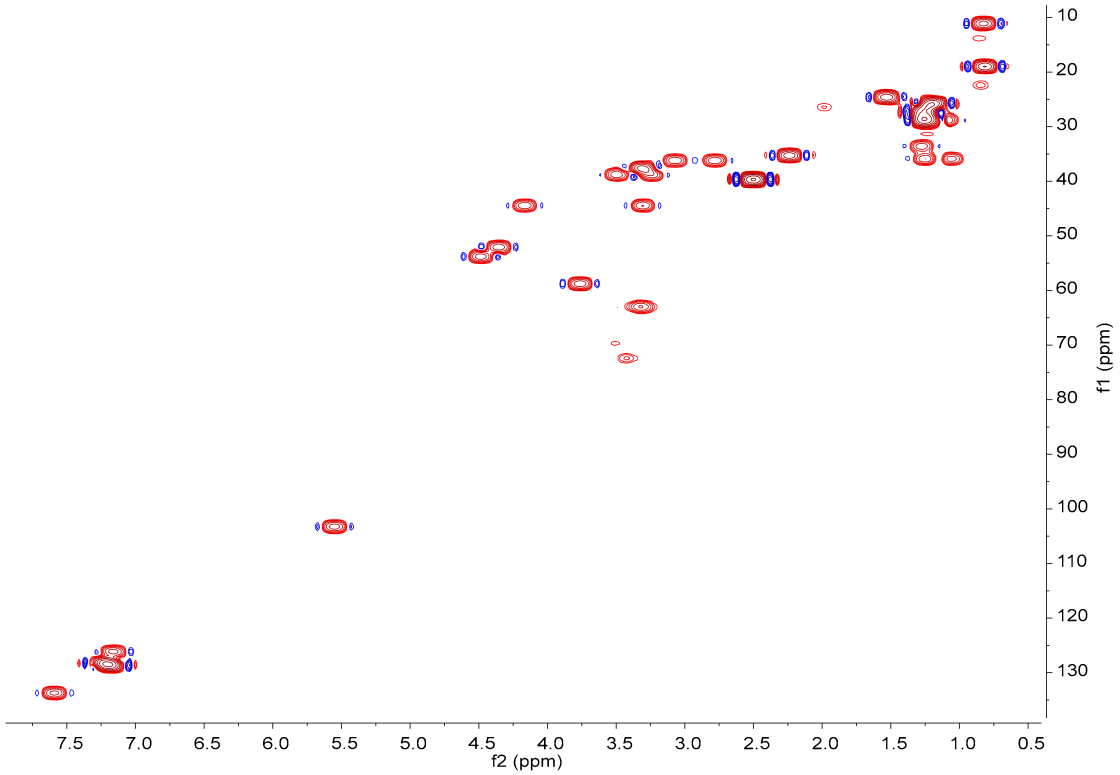

Figure S9. HSQC spectrum of **1** in DMSO-d_6.

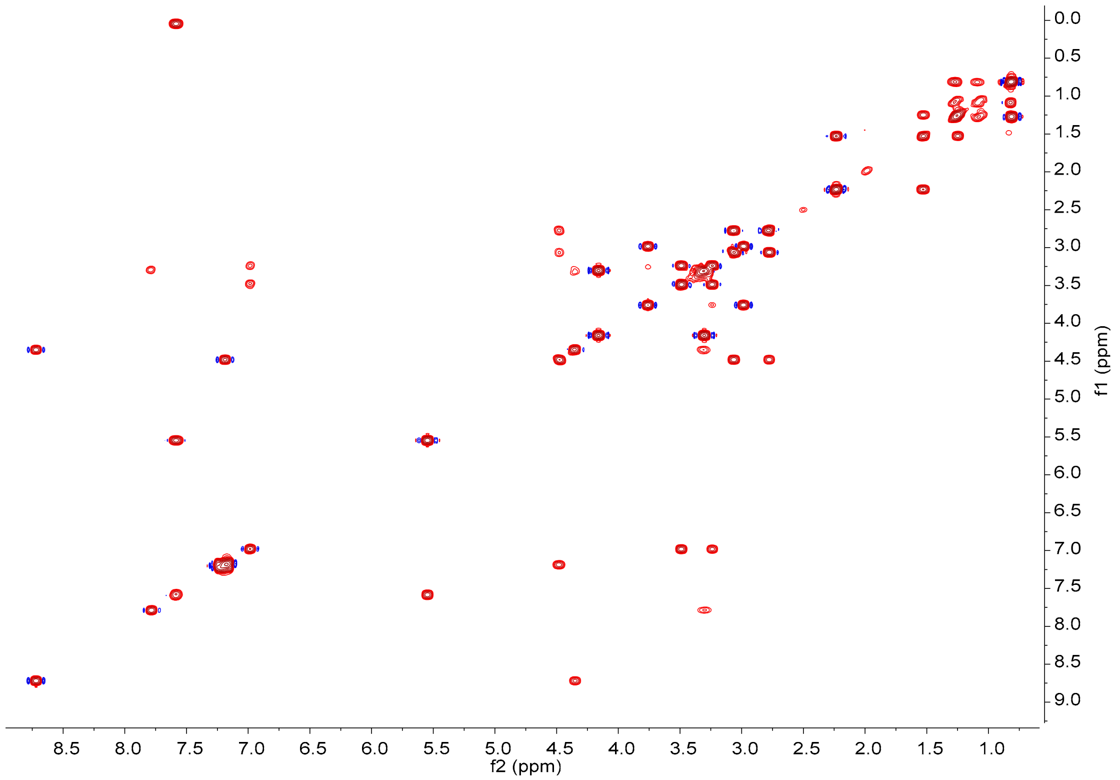

Figure S10. ^{1}H-^{1}H COSY spectrum of **1** in DMSO-*d*$_6$.

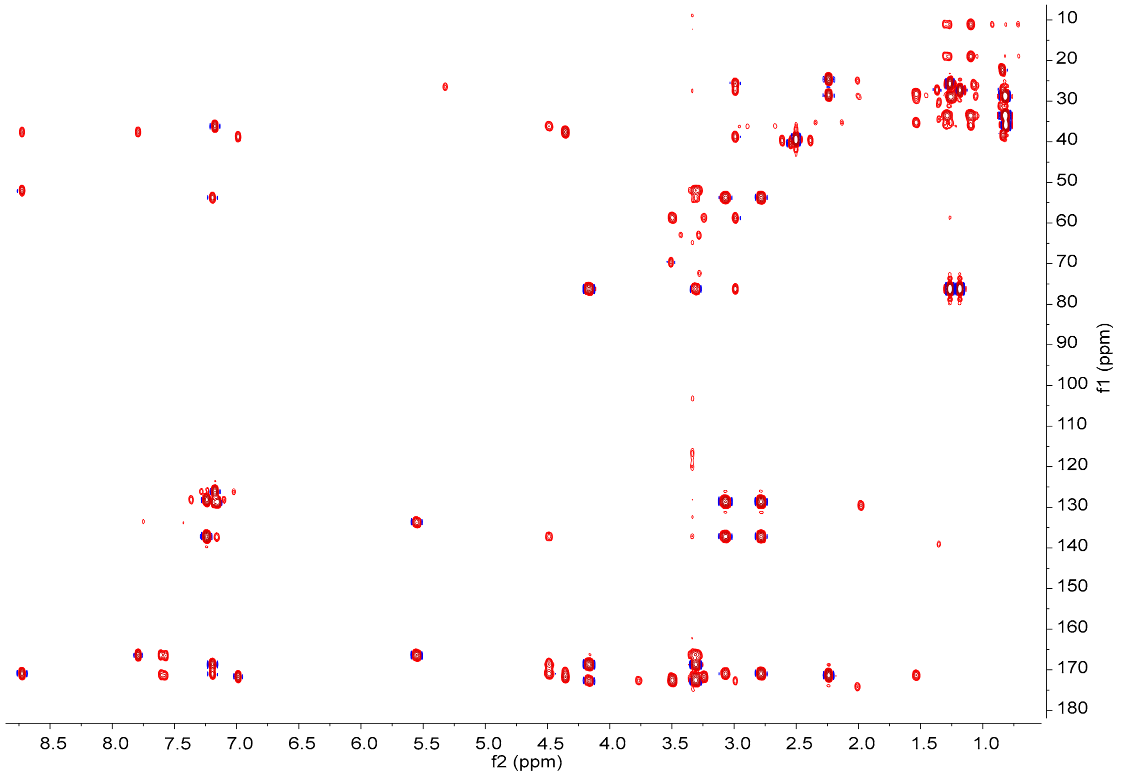

Figure S11. HMBC spectrum of **1** in DMSO-*d*$_6$.

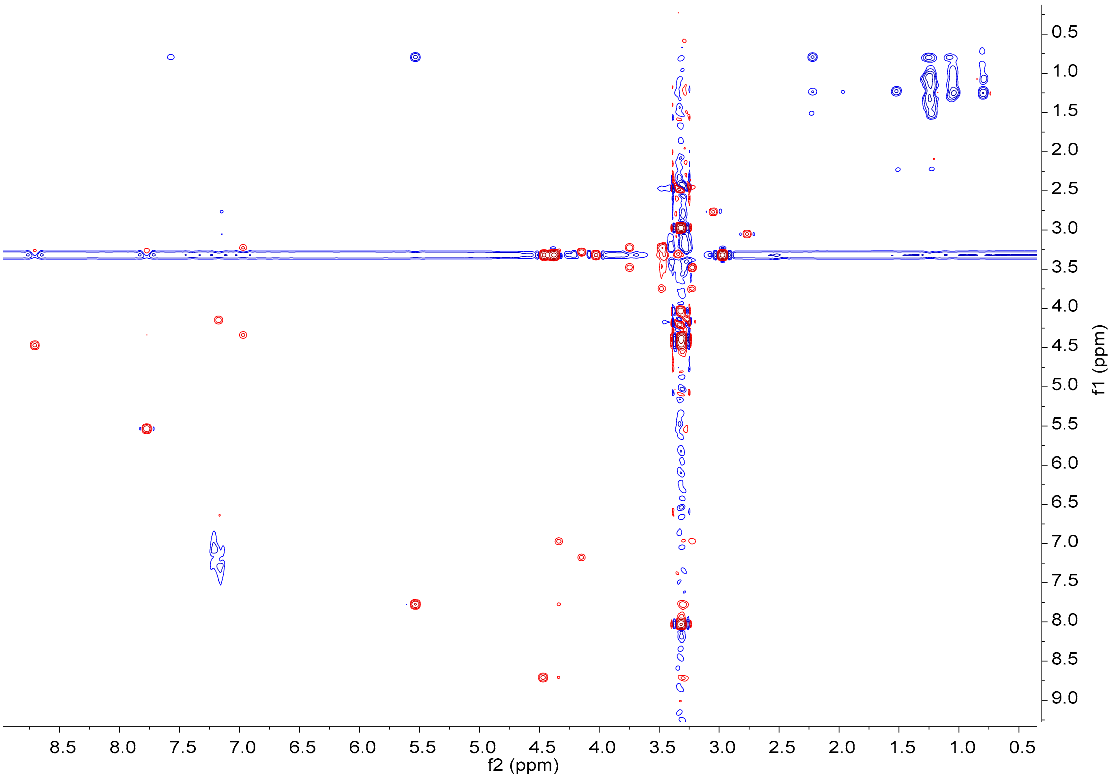

Figure S12. NOESY spectrum of **1** in DMSO-d_6.

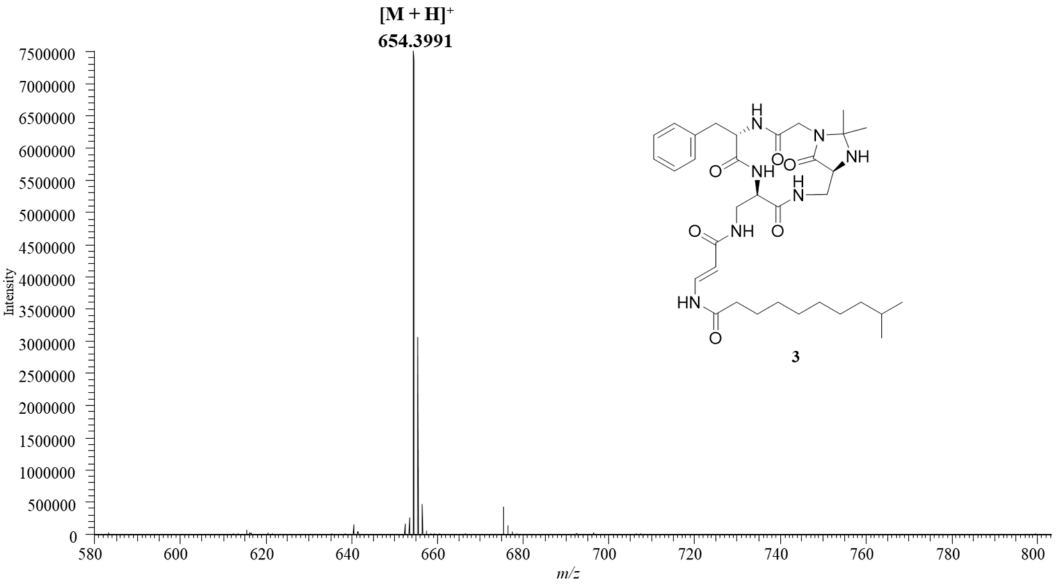

Figure S13. HRESIMS spectrum of **2**.

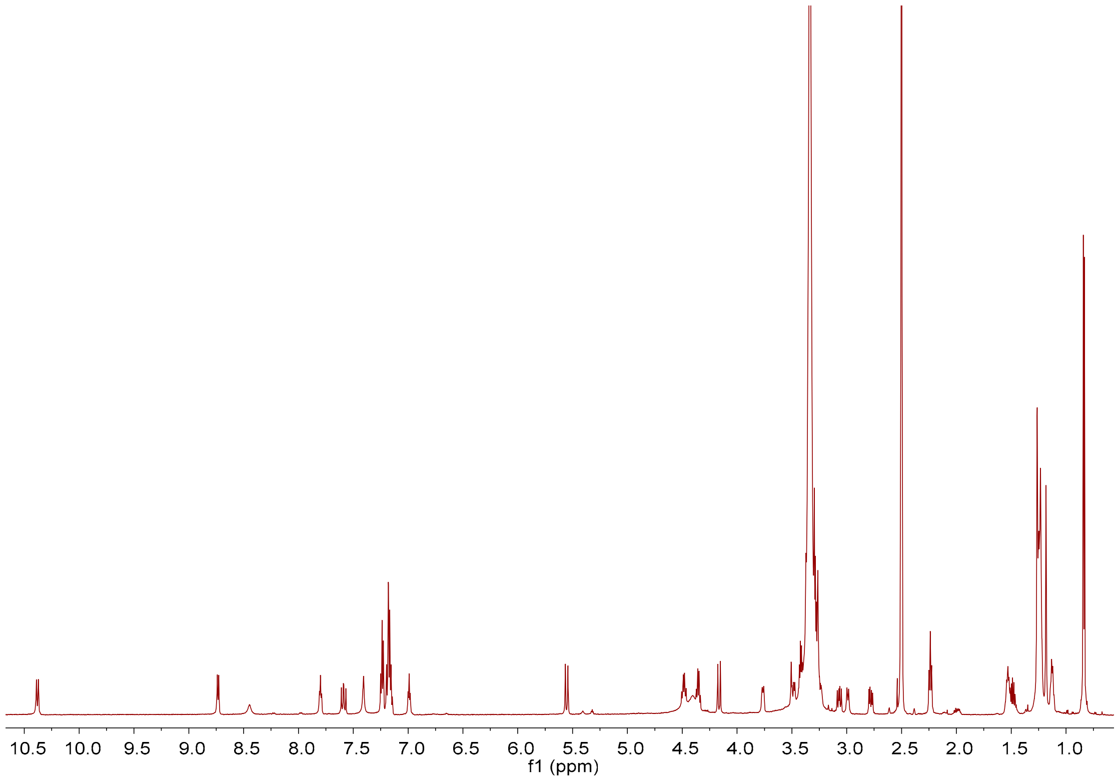

Figure S14. ^{1}H NMR spectrum of **2** in DMSO-*d₆*.

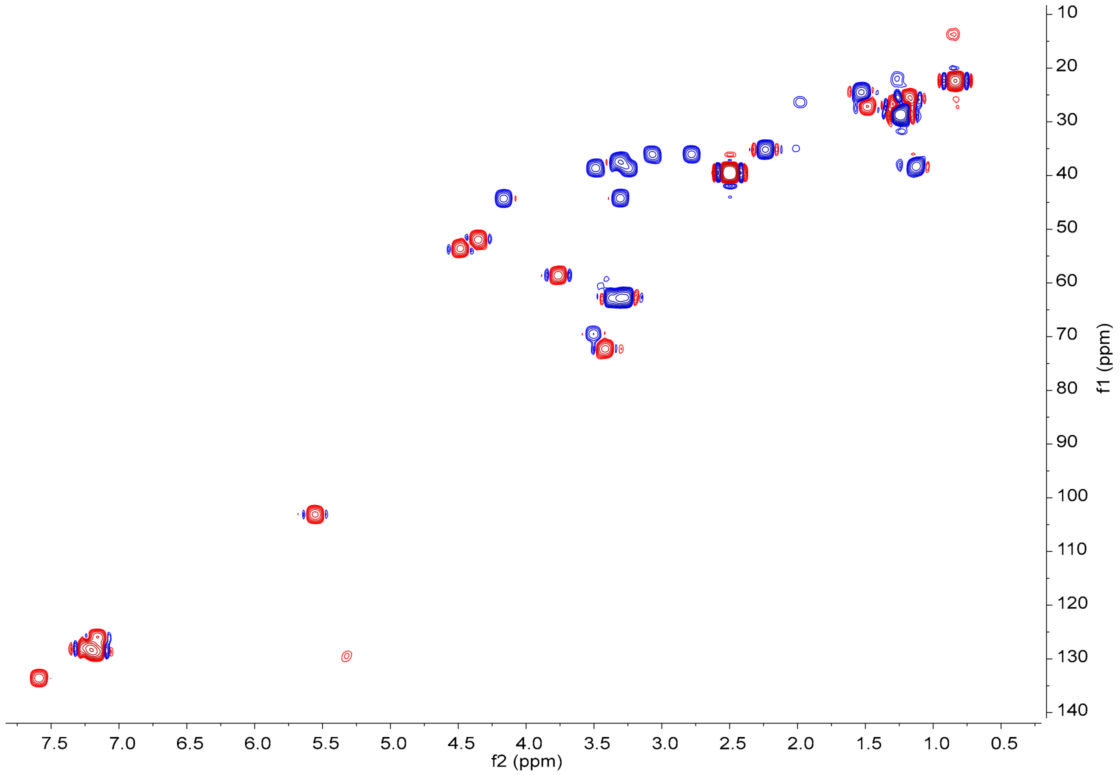

Figure S15. HSQC spectrum of **2** in DMSO-*d₆*.

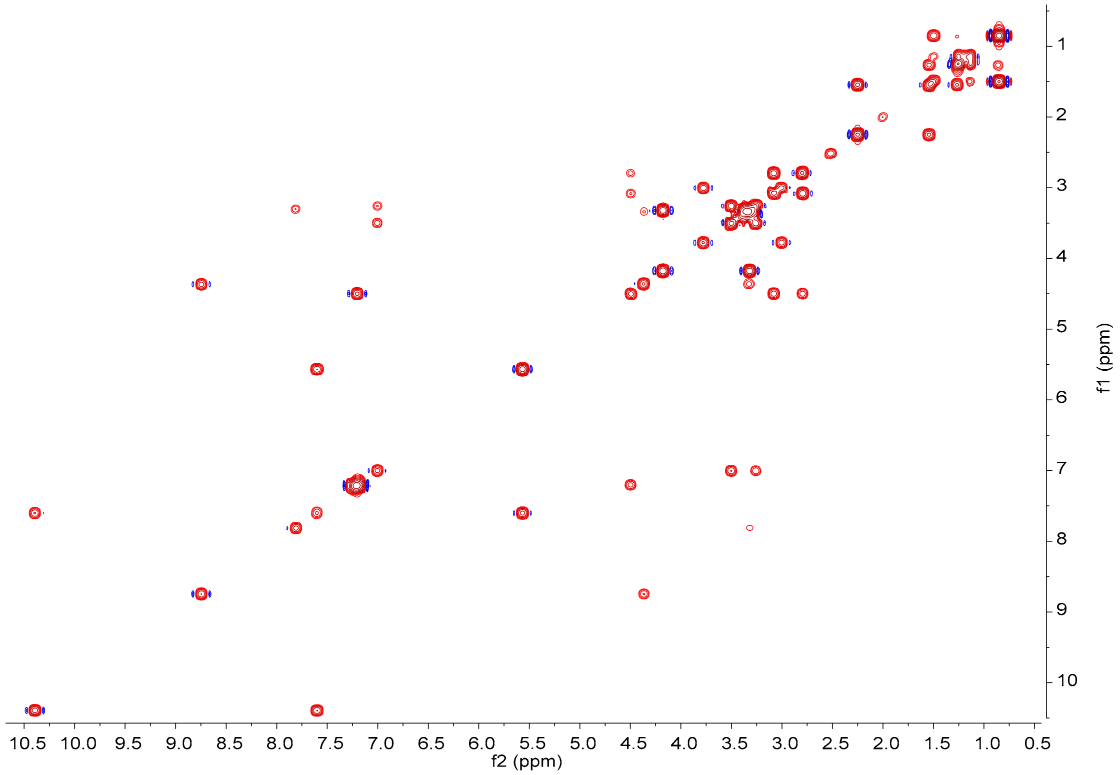

Figure S16. ^{1}H-^{1}H COSY spectrum of **2** in DMSO-d_6.

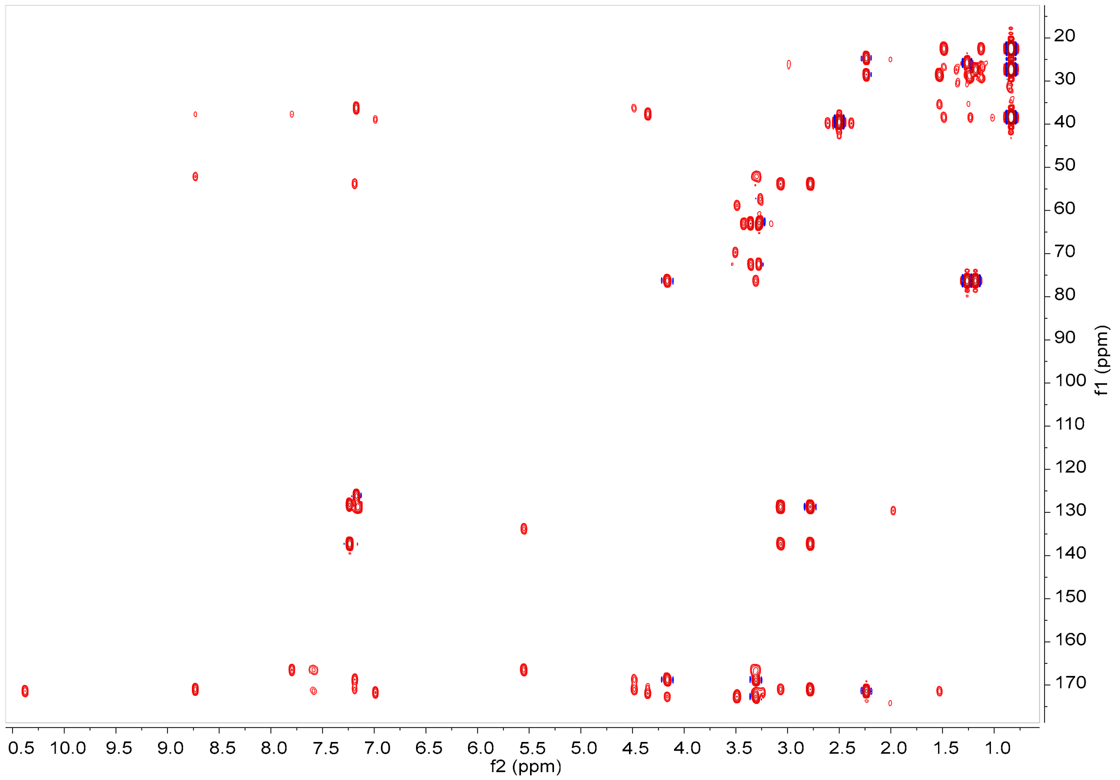

Figure S17. HMBC spectrum of **2** in DMSO-d_6.

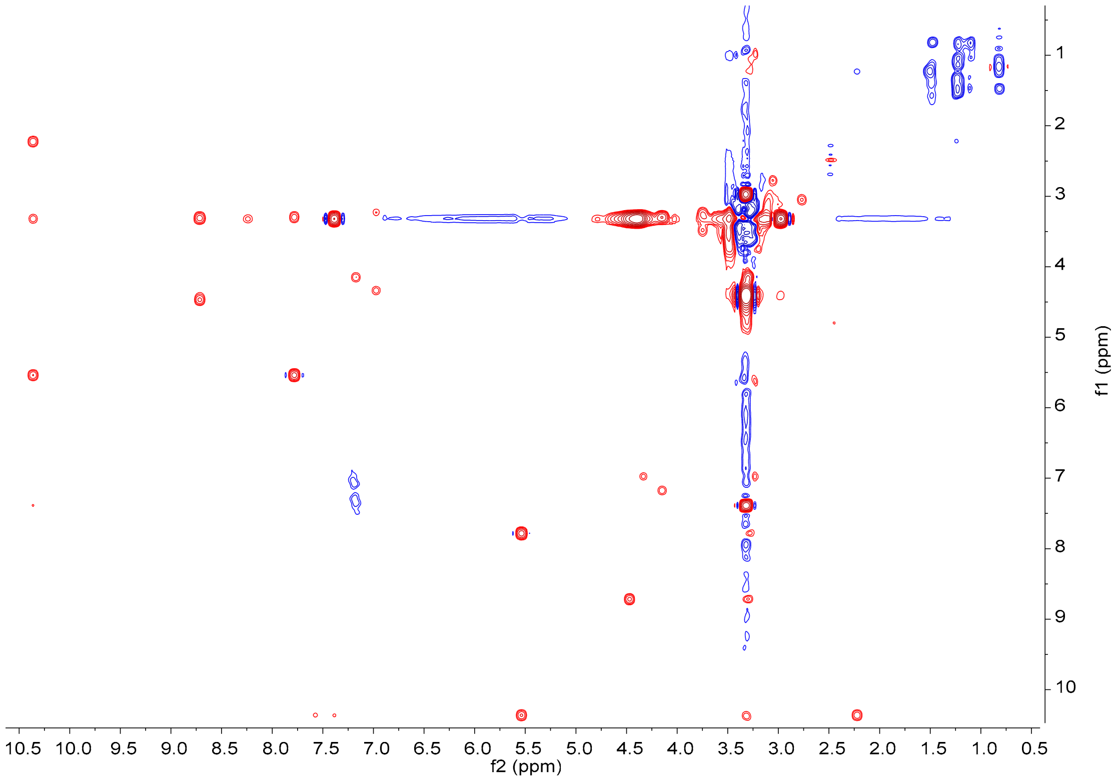

Figure S18. NOESY spectrum of **2** in DMSO-*d*₆.

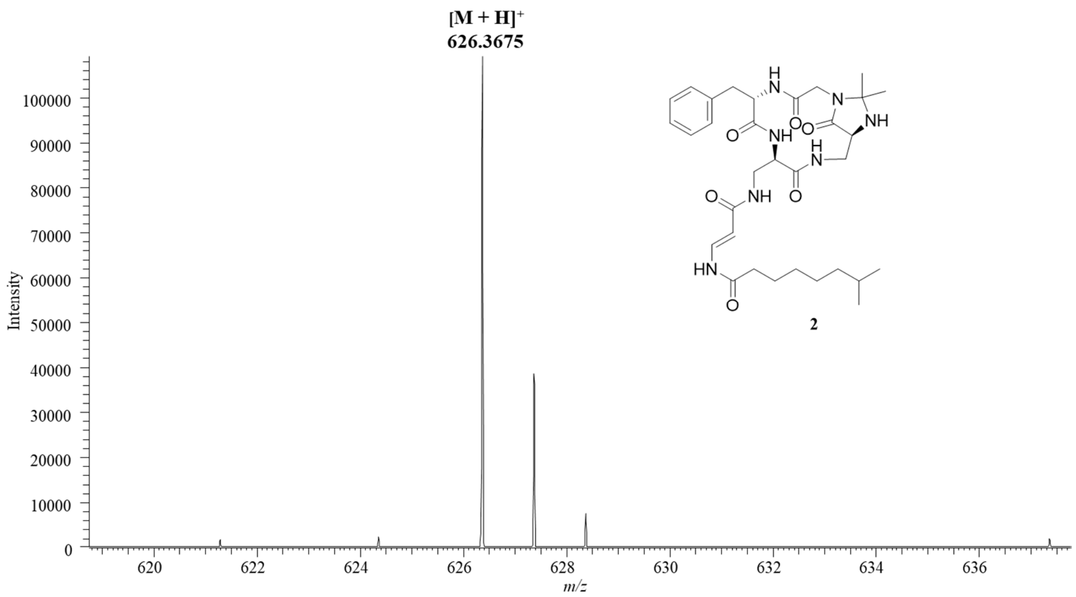

Figure S19. HRESIMS spectrum of **3**.

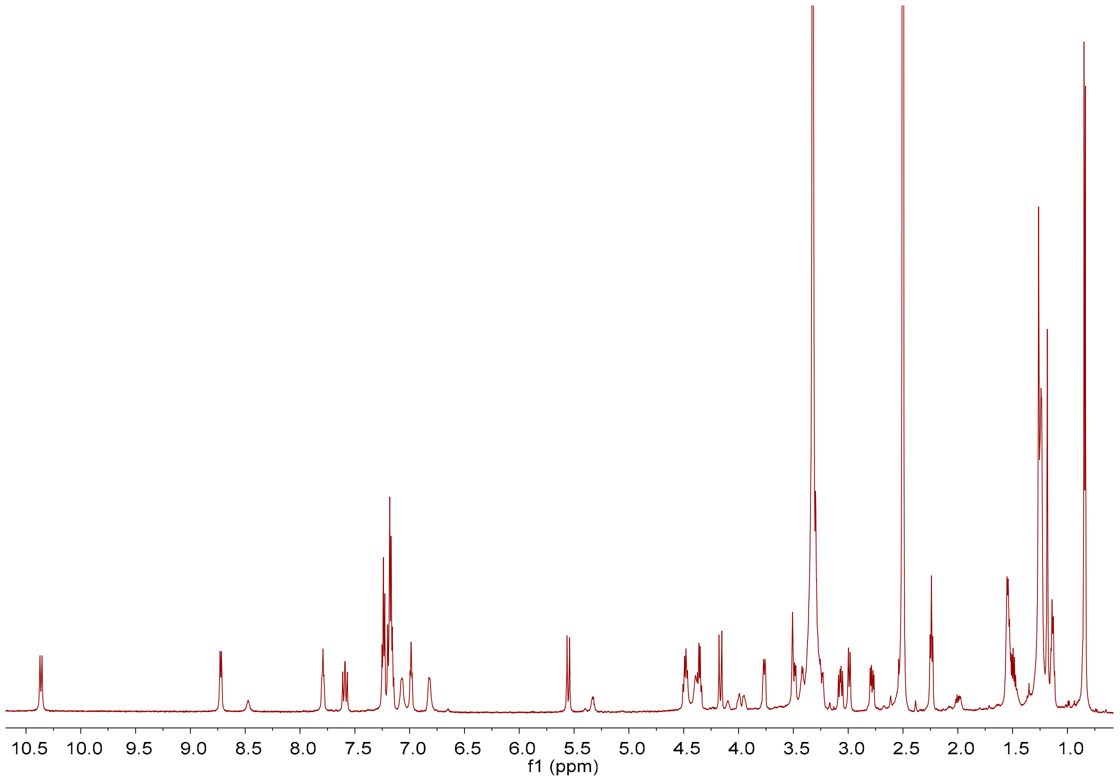

Figure S20. ^{1}H NMR spectrum of **3** in DMSO-*d*6.

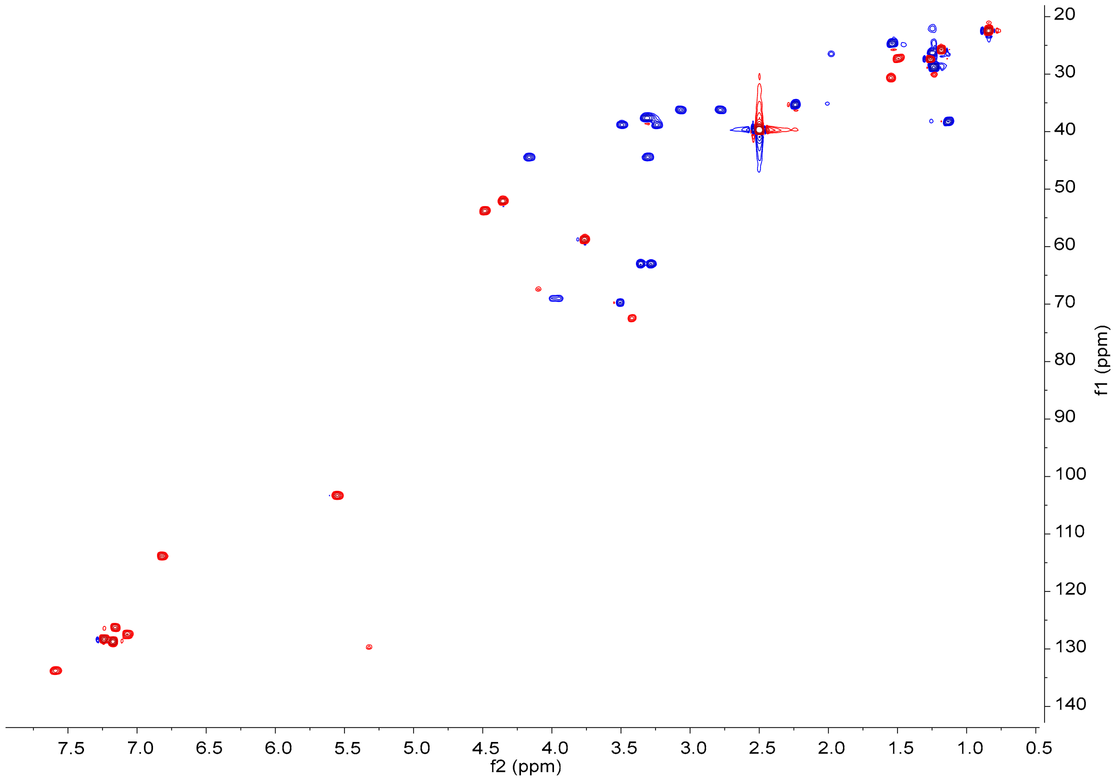

Figure S21. HSQC spectrum of **3** in DMSO-*d*6.

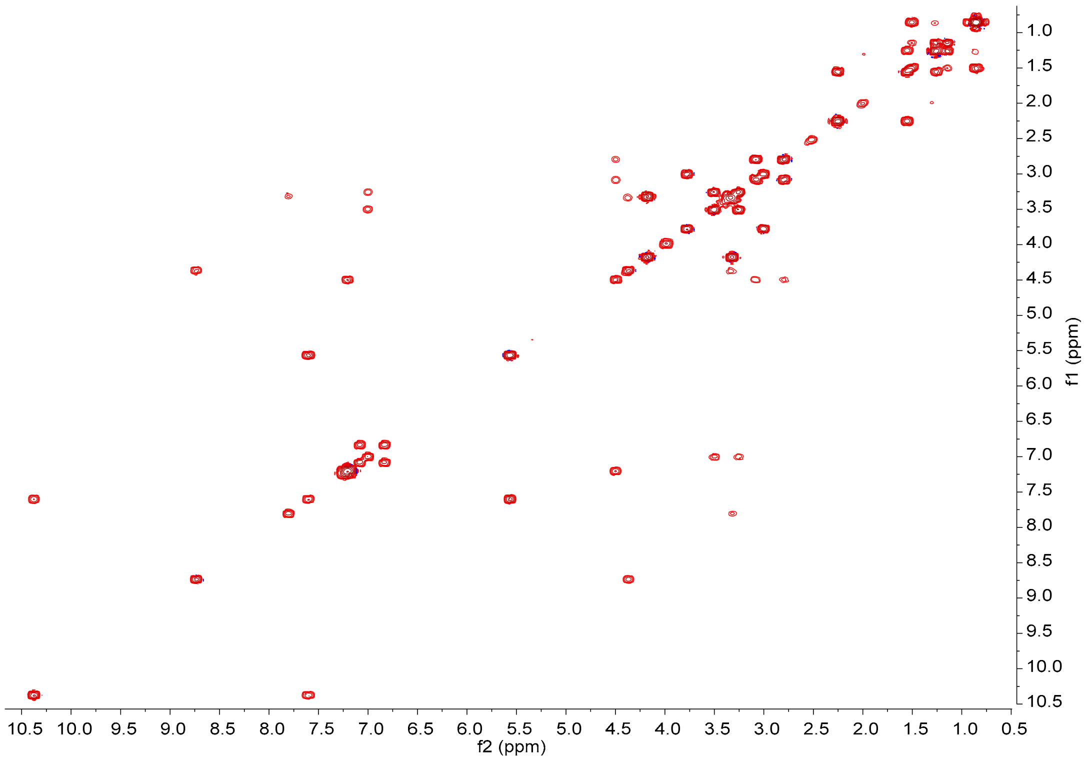

Figure S22. ^{1}H-^{1}H COSY spectrum of **3** in DMSO-*d*6.

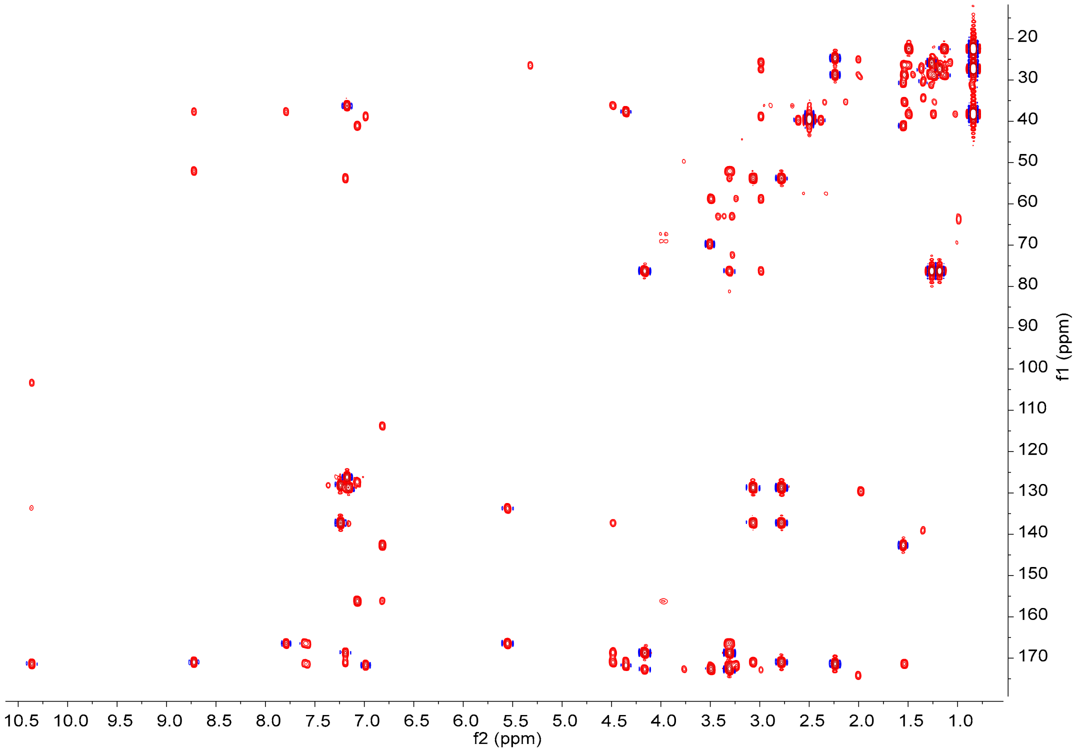

Figure S23. HMBC spectrum of **3** in DMSO-*d*6.

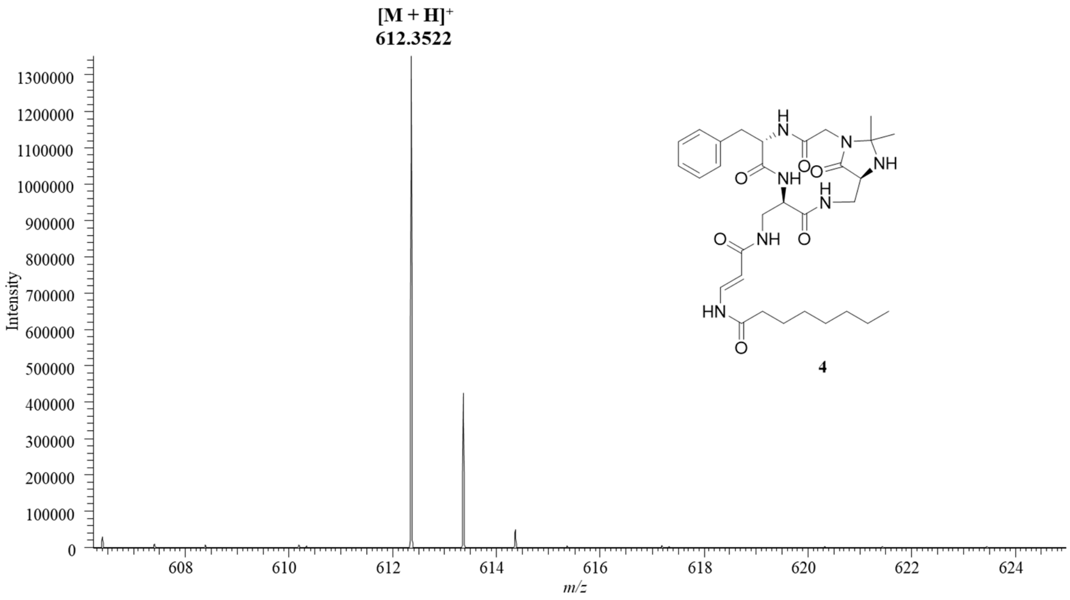

Figure S24. HRESIMS spectrum of **4**.

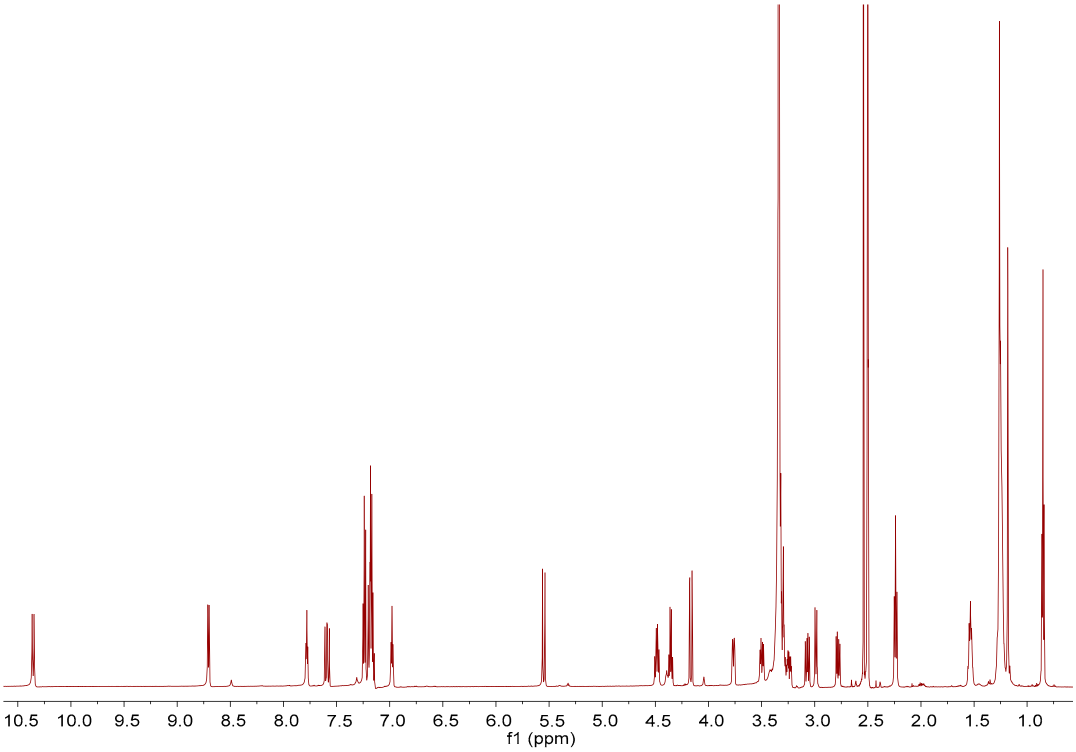

Figure S25. ^{1}H NMR spectrum of **4** in DMSO-d_6.

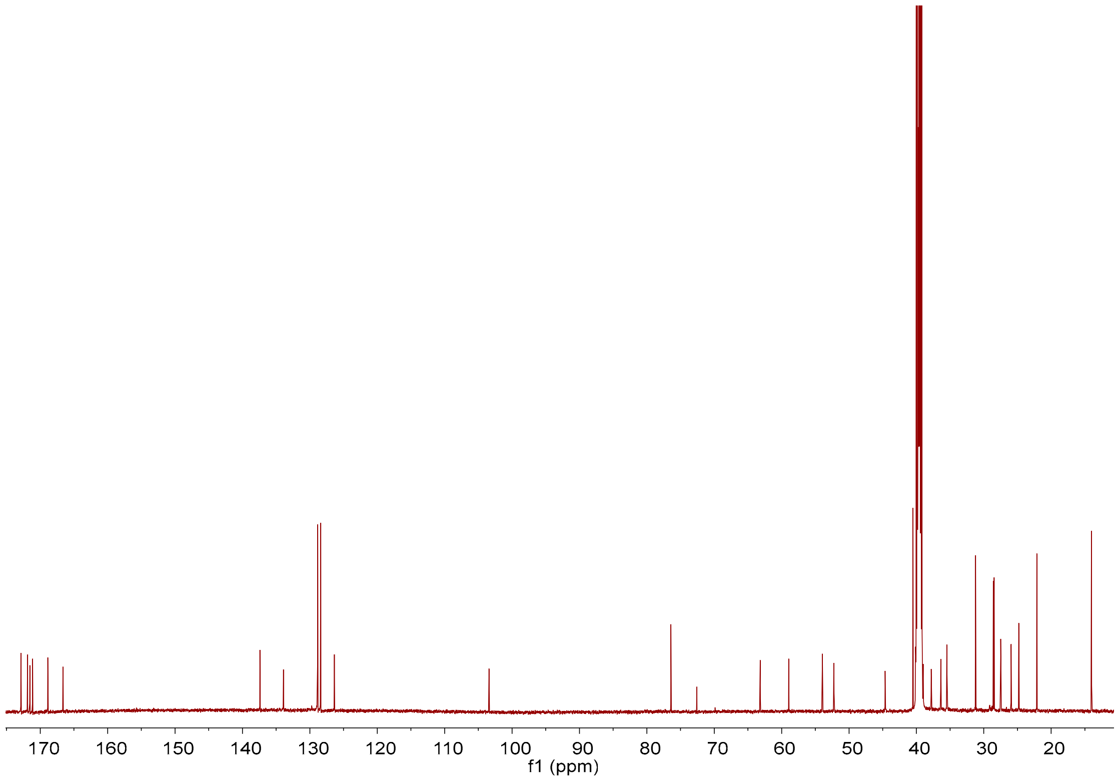

Figure S26. ^{13}C NMR spectrum of **4** in DMSO-*d*$_6$.

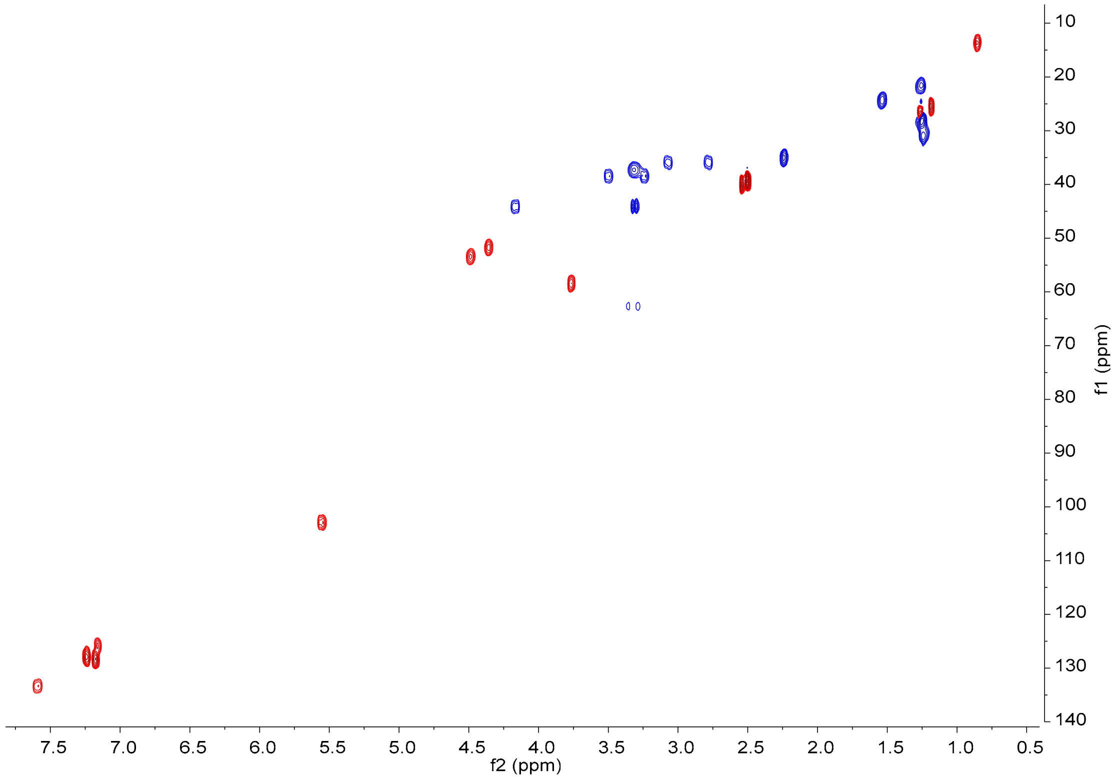

Figure S27. HSQC spectrum of **4** in DMSO-*d*$_6$.

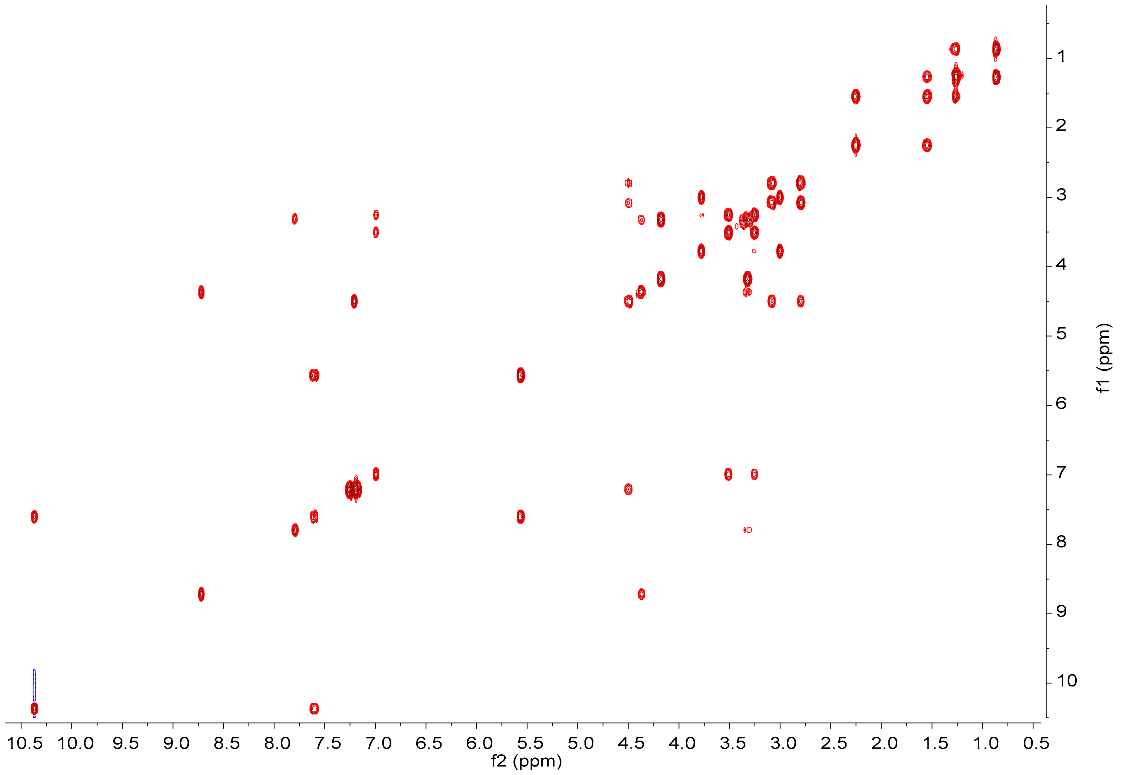

Figure S28. ^{1}H-^{1}H COSY spectrum of **4** in DMSO-*d*6.

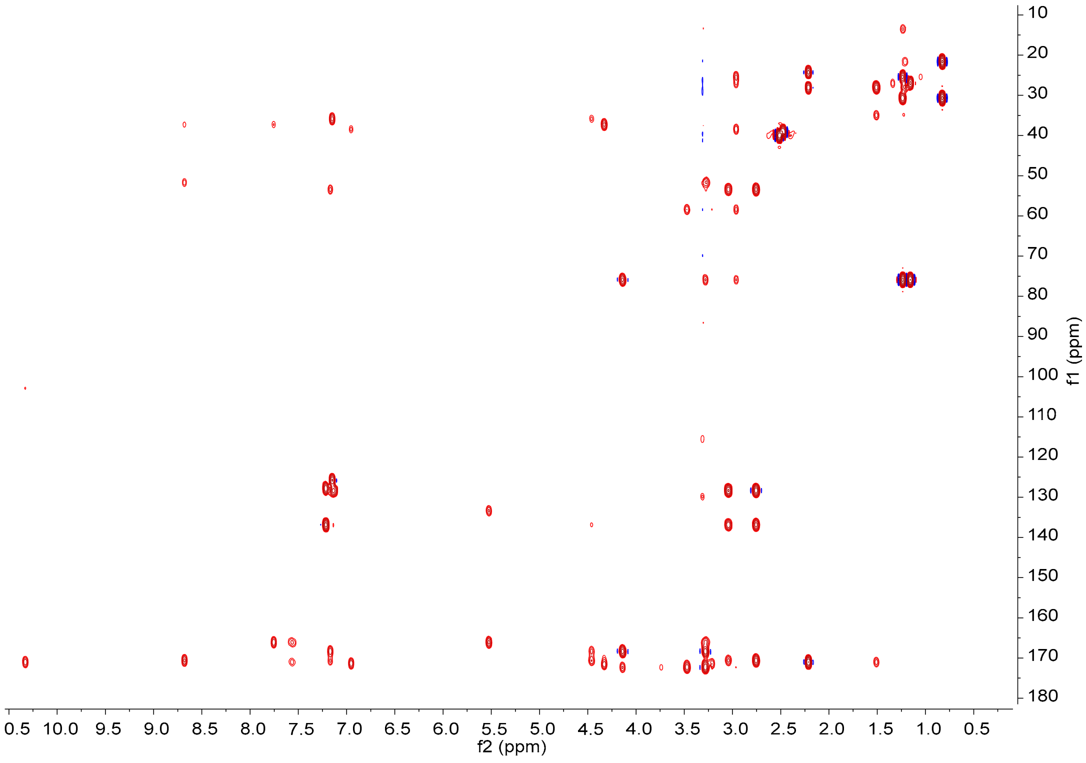

Figure S29. HMBC spectrum of **4** in DMSO-*d*6.

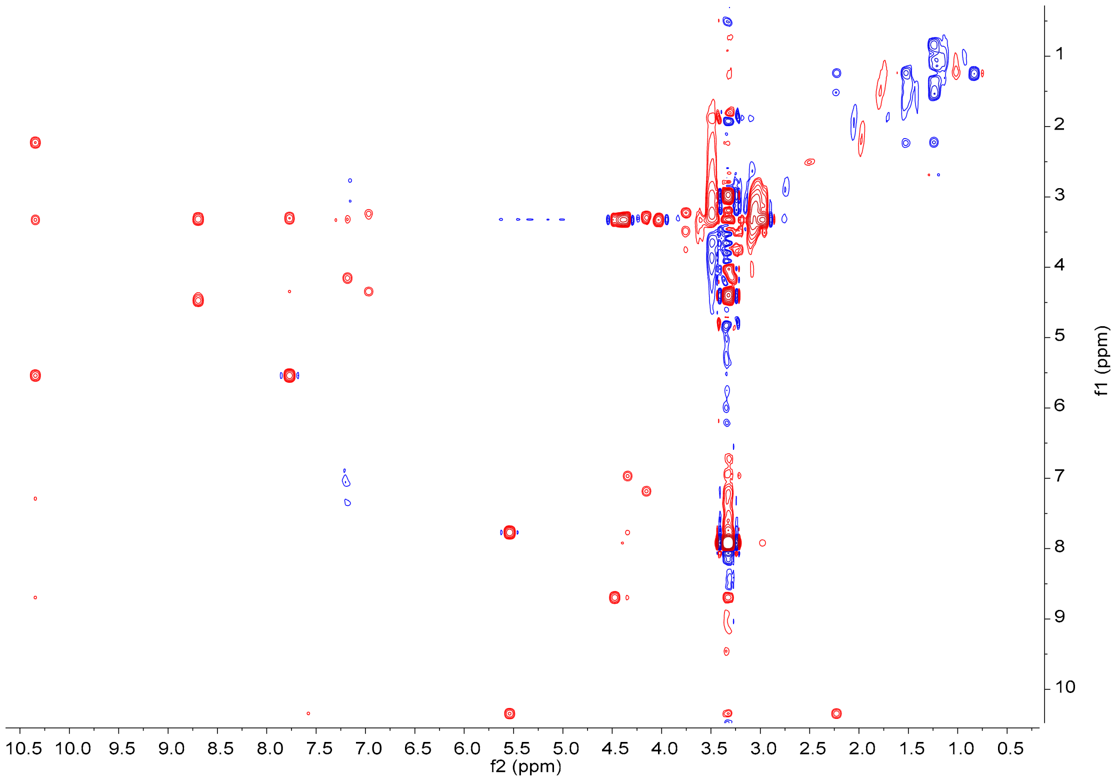

Figure S30. NOESY spectrum of **4** in DMSO-d_6.

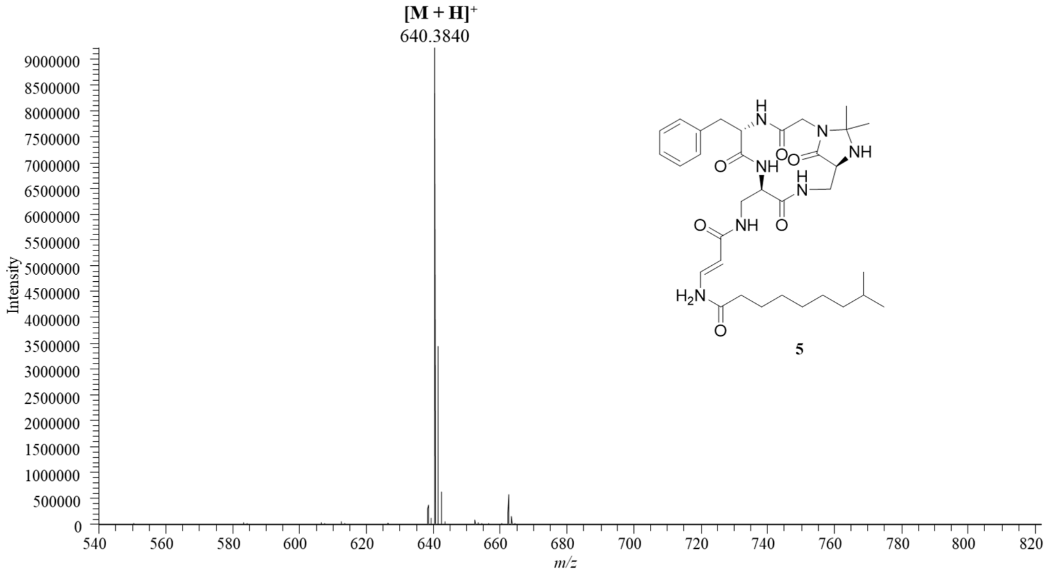

Figure S31. HR-ESIMS spectrum of **5**.

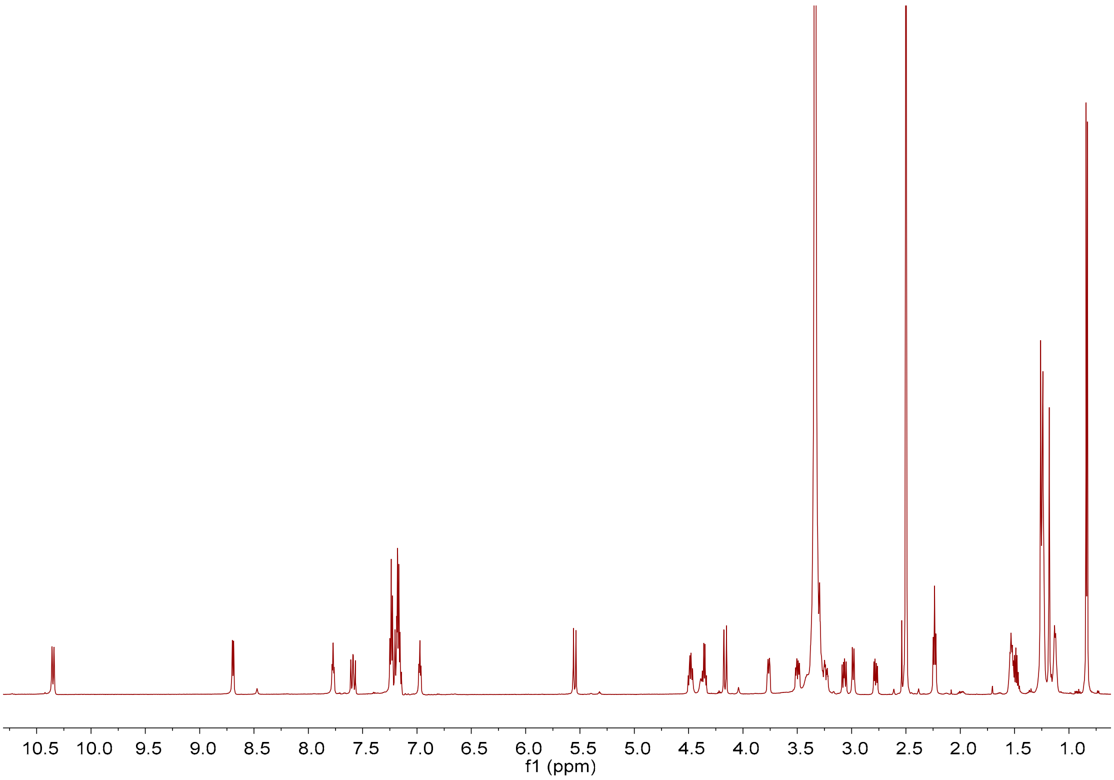

Figure S32. ^{1}H NMR spectrum of **5** in DMSO-d_6.

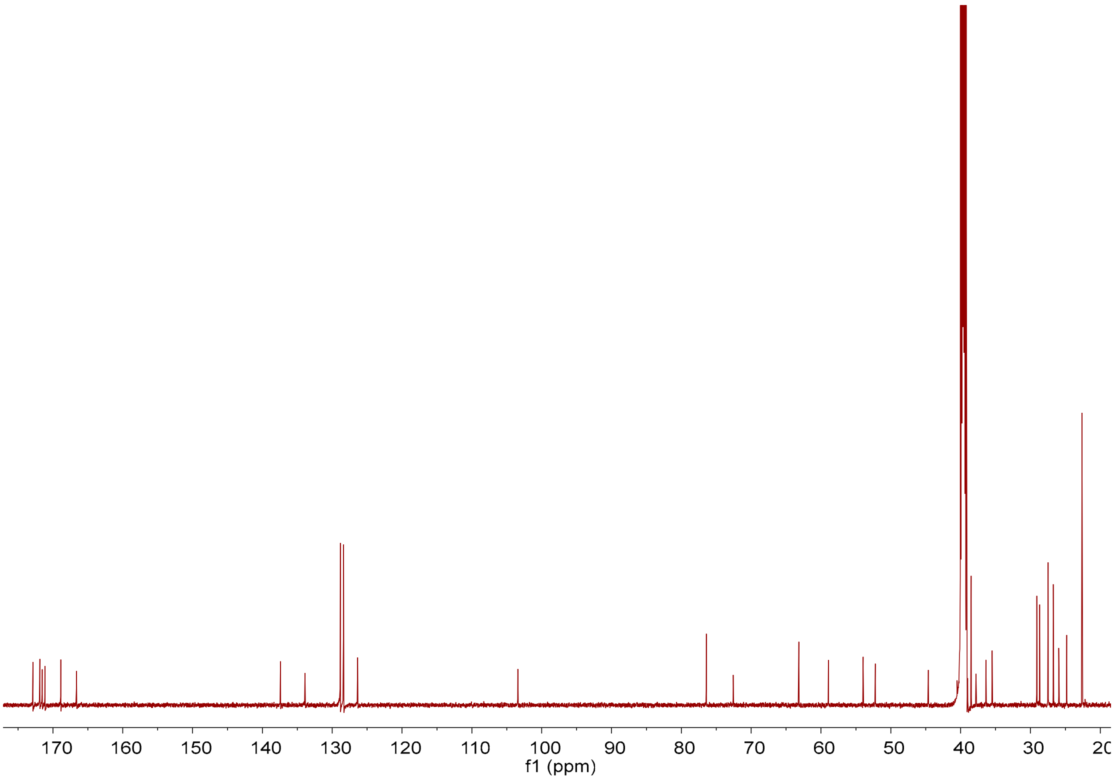

Figure S33. ^{13}C NMR spectrum of **5** in DMSO-d_6.

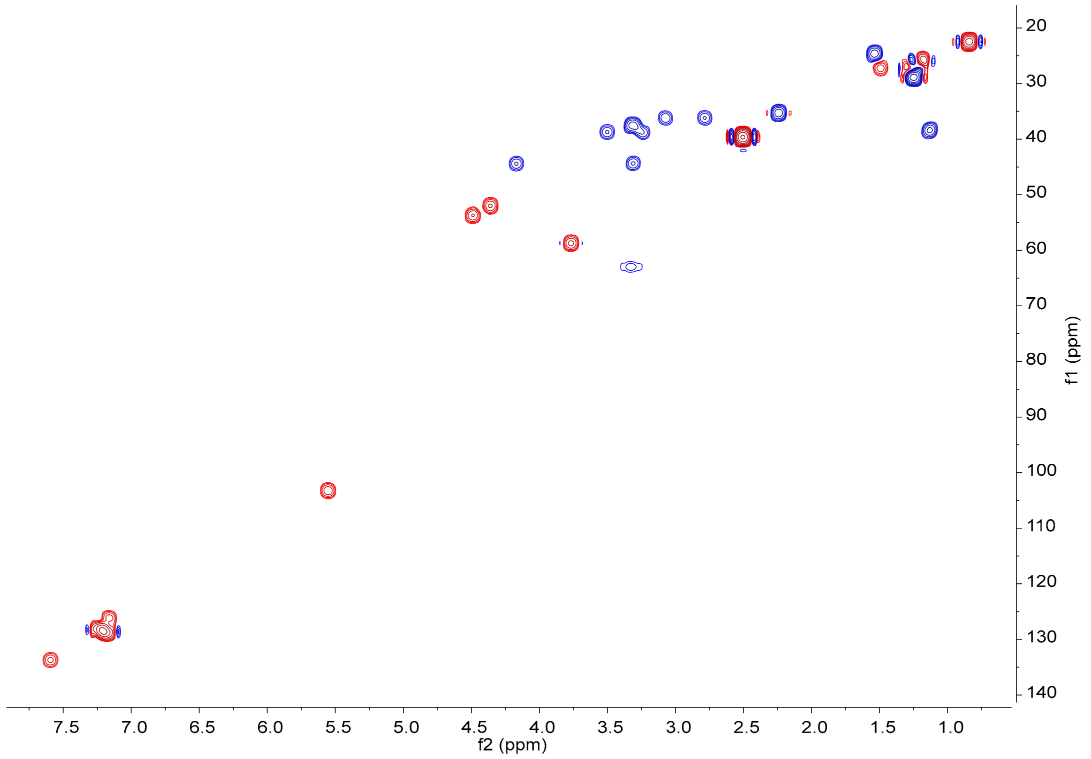

Figure S34. HSQC spectrum of **5** in DMSO-d_6.

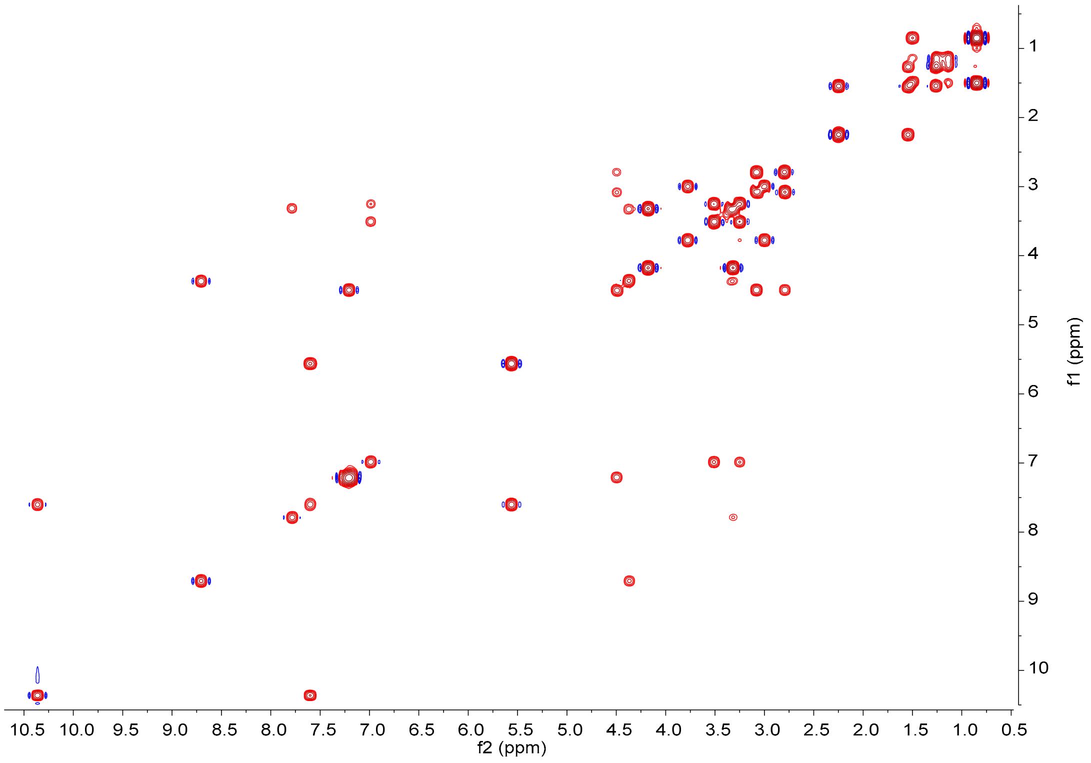

Figure S35. ^{1}H-^{1}H COSY spectrum of **5** in DMSO-d_6.

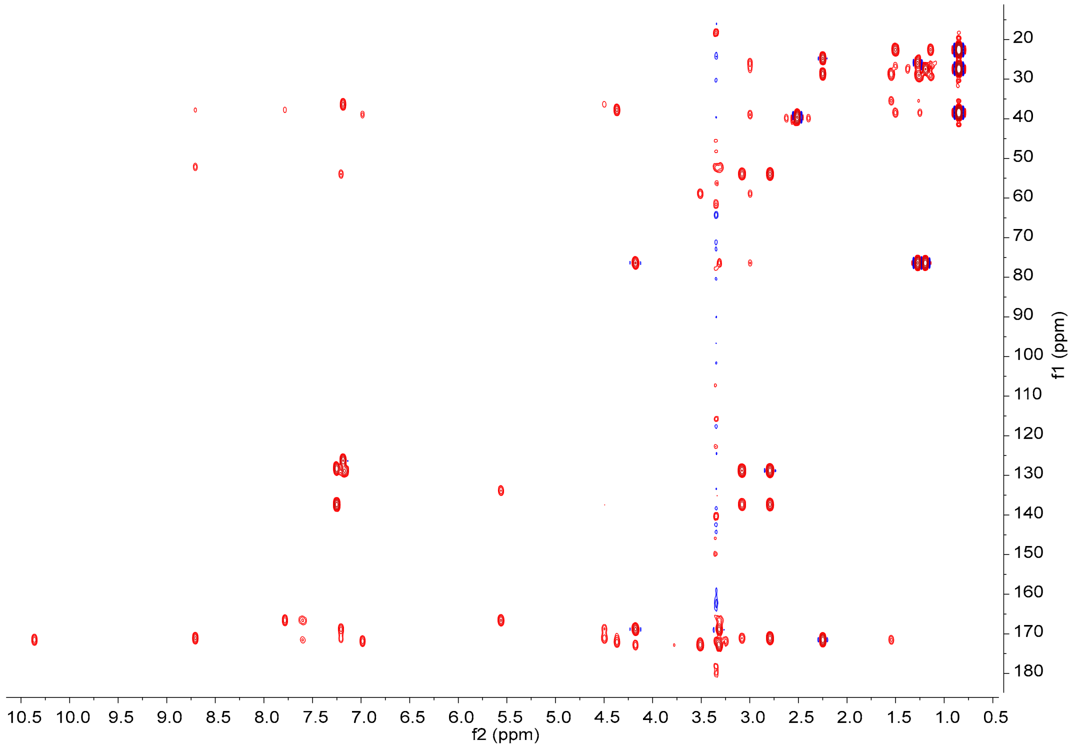

Figure S36. HMBC spectrum of **5** in DMSO-*d*$_6$.

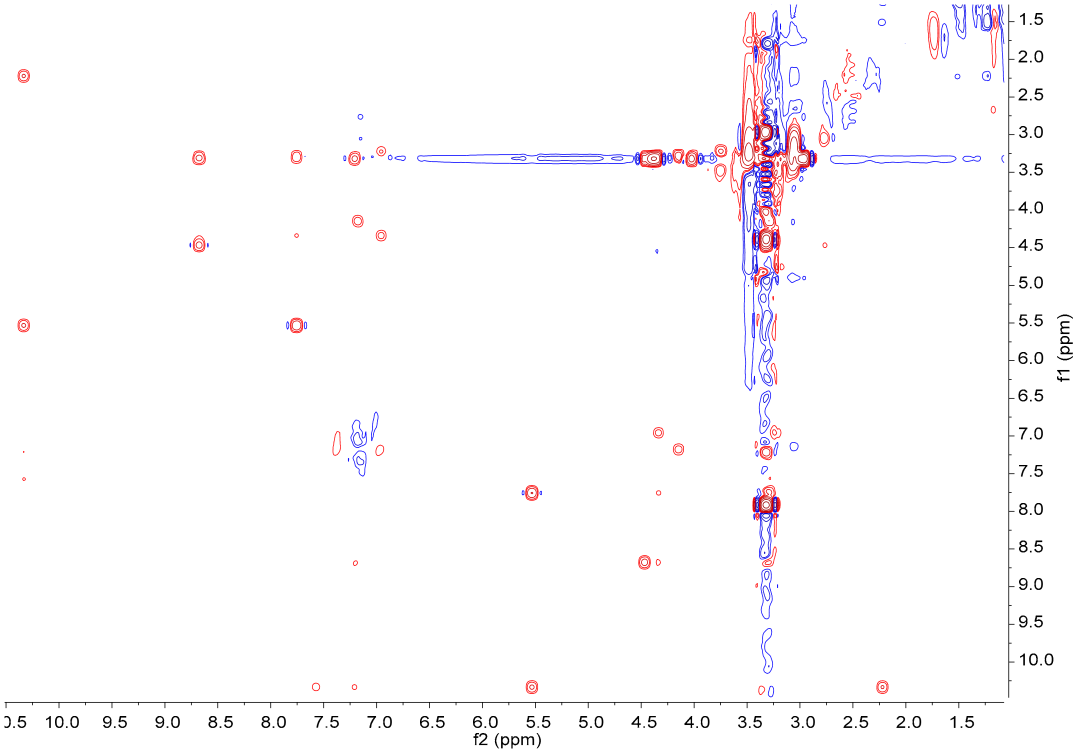

Figure S37. NOESY spectrum of **5** in DMSO-*d*$_6$.

References

[1] Gust B, Challis G L, Fowler K, Kieser T, Chater K F. PCR-targeted Streptomyces gene replacement identifies a protein domain needed for biosynthesis of the sesquiterpene soil odor geosmin[J]. *Proc Natl Acad Sci U S A*, 2003, 100(4): 1541-1546.

[2] Macneil D J, Occi J L, Gewain K M, Macneil T, Gibbons P H, Ruby C L, Danis S J. Complex organization of the *Streptomyces avermitilis* genes encoding the avermectin polyketide synthase[J]. *Gene*, 1992, 115(1-2): 119-125.
